# Sec8: a novel positive regulator of RIG-I in anti-RNA viral defense

**DOI:** 10.1038/s41419-026-08414-9

**Published:** 2026-01-24

**Authors:** Lin Wang, Wenqing Ma, Peili Hou, Rong Jin, Xinxin Wei, Xingyu Li, Daniel Chang He, Hongmei Wang, Hongbin He

**Affiliations:** 1 Ruminant Diseases Research Center, College of Life Sciences, Shandong Normal University, Jinan, Shandong China; 2 The College of Arts and Sciences, University of North Carolina at Chapel Hill, Chapel Hill, NC USA

**Keywords:** RIG-I-like receptors, Ubiquitylation

## Abstract

Sec8, an exocyst complex subunit, is pivotal in facilitating the docking of exocytic vesicles to fusion sites on the plasma membrane. However, its involvement in the antiviral innate immune response and virus replication remains unclear. In this study, Sec8 is identified as a novel positive regulator of RIG-I, enhancing the IFN-I signaling response against RNA viruses both in vivo and in vitro. Additionally, Sec8 stabilizes RIG-I by inhibiting its ubiquitination and subsequent proteasome-mediated degradation. Mechanistically, STUB1 degrades RIG-I via K48-linked ubiquitination at Lys190, while Sec8 suppresses STUB1 mRNA by reducing the expression of p53 and competes with STUB1 for binding to RIG-I’s CARD domain, thereby preventing STUB1-mediated RIG-I degradation. Importantly, Sec8-deficient mice were more susceptible to RNA virus infection compared to wild-type mice. These findings elucidate a mechanism that Sec8 positively regulates RIG-I in the antiviral innate immune response, offering insights for developing novel therapeutic strategies and targeted antiviral medications.

## Introduction

Over the recent past, viral infections have posed significant threats to the well-being of humans and animals. Pattern recognition receptors (PRRs) activate signaling cascades by detecting different pathogen-associated molecular patterns (PAMPs), leading to the up-regulation of numerous immune genes, including type I interferons, inflammatory cytokines, and chemokines, enabling the host to establish appropriate innate immunity [1, 2]. Retinoic acid-inducible gene I (RIG-I), a pattern recognition receptor (PRR), plays an indispensable role in triggering the innate immune response against RNA viral infections [3, 4]. This large multi-structural domain protein undergoes conformational changes upon viral infection, revealing two structural domains of the caspase activation and recruitment domains (CARDs) that acquire an active state by selectively recognizing and binding to 5′-triphosphate blunt-ended RNA (5′-pppRNA) and short dsRNA [5]. Subsequently, the TBK1-IRF3 signaling cascade is triggered by activated RIG-I interacting with mitochondrial antiviral signaling protein (MAVS), which results in the generation of type I interferon (IFN-I) [6]. The JAK/STAT signaling pathway is then activated by IFN-I, leading to a massive production of IFN-stimulated genes (ISGs), which are essential for the intracellular antiviral defense mechanism and immunological control. Precise control of RIG-I signaling is required for the host to respond to foreign pathogens and to prevent autoimmune-related diseases caused by over-activation [7].

RIG-I is one of the most important promoters of RIG-I-like receptor (RLR) signaling in the sense of RNA viruses, and it has been well-established as a key player in the host’s innate defense against RNA virus infection and in maintaining immunological homeostasis. The ubiquitination modification of RIG-I is essential for the immune signaling. Multiple factors have been shown to modulate RIG-I at the protein level, either positively or negatively, through various mechanisms [8]. Positive modulators, including tripartite motif-containing protein 4 (TRIM4), tripartite motif-containing protein 25 (TRIM25), mex-3 RNA binding family member C (Mex3c), and rING finger protein 135 (RNF135/Riplet) [9–12], mediate K63-linked ubiquitination of RIG-I to promote RIG-I activation and initiate the production of antiviral type I IFNs. To maintain immune homeostasis and prevent excessive inflammation, RIG-I activity is carefully controlled by a range of negative regulators through diverse molecular strategies. Deubiquitinating enzymes such as cylindromatosis (CYLD) and ubiquitin-specific peptidase 21 (USP21) can remove the critical K63-linked ubiquitin chain from RIG-I, thereby deactivating it and interrupting signal transduction [13–15]. E3 ubiquitin ligases like ring finger protein 125 (RNF125), tripartite motif-containing protein 40 (TRIM40), STIP1 homology and U-Box containing protein 1 (STUB1), and casitas B-lineage lymphoma (c-Cbl) target RIG-I for degradation via the proteasome pathway by attaching K48-linked ubiquitin chains [7, 16–19]. NLR family CARD domain containing 5 (NLRC5) directly inhibits downstream signaling by competing with RIG-I for binding to the MAVS signaling complex on mitochondria [20]. Sialic acid-binding immunoglobulin-like lectin-G (Siglec-G) recruits phosphatase SHP2 to dephosphorylate RIG-I, which requires phosphorylation for activation, leading to an inhibitory effect on RIG-I [18]. Although these advances in delineating the regulatory network of RIG-I are significant, whether additional host factors exist that modulate RIG-I stability by targeting these key regulatory molecules remains to be explored.

The exocyst complex is an evolutionarily conserved octameric protein complex composed of Secretory 3 (Sec3), Secretory 5 (Sec5), Secretory 6 (Sec6), Secretory 8 (Sec8), Secretory 10 (Sec10), Secretory 15 (Sec15), exocyst complex 70 KDa subunit (EXO70), and exocyst complex 84 KDa subunit (EXO84) [21]. The exocyst is widely expressed in various species and plays a crucial role in cell polarization, ciliogenesis, autophagy, host defense, and tumor invasion [22]. Individual subunits of the exocyst play their distinct roles in influencing antiviral natural immunity or viral replication. Sec3 has been reported to act as a negative regulator of flavivirus RNA transcription and translation through elongation factor-α (EF1α) [23]; Furthermore, Sec5 enhances SARS-CoV-2 infection by downregulating interferon omega-1 (IFNW1) expression [24]; Sec6 enhances the replication of the Singapore grouper iridovirus (SGIV) by deregulating the promoters of nuclear factor kappa-B (NF-κB) and activator protein-1(AP-1) [25]. Sec8, also known as exocyst complex component 4 (EXOC4), is highly conserved in eukaryotes. The Sec8 gene generates a 110 kDa multi-domain protein containing 974 amino acids. It has been reported to be involved in the tethering of secretory vesicles to the plasma membrane [26]. In addition, Sec8 has been shown to play a role in a variety of cancers, including cervical [27], lung [28], gastric [29], and prostate cancers [30]. Nevertheless, the role of Sec8 in antiviral innate immunity and defense against viruses remains unclear.

In the study, we first demonstrated that Sec8 inhibits STUB1-mediated RIG-I degradation, which promotes IFN-I response and inhibits viral replication in vitro and in vivo animal models. Our work reveals a previously unknown function of Sec8 in positively regulating the innate immune response to viral infection.

## Results and discussion

### Sec8 positively regulates RNA virus-induced IFN-I signaling

**Fig. 1:**
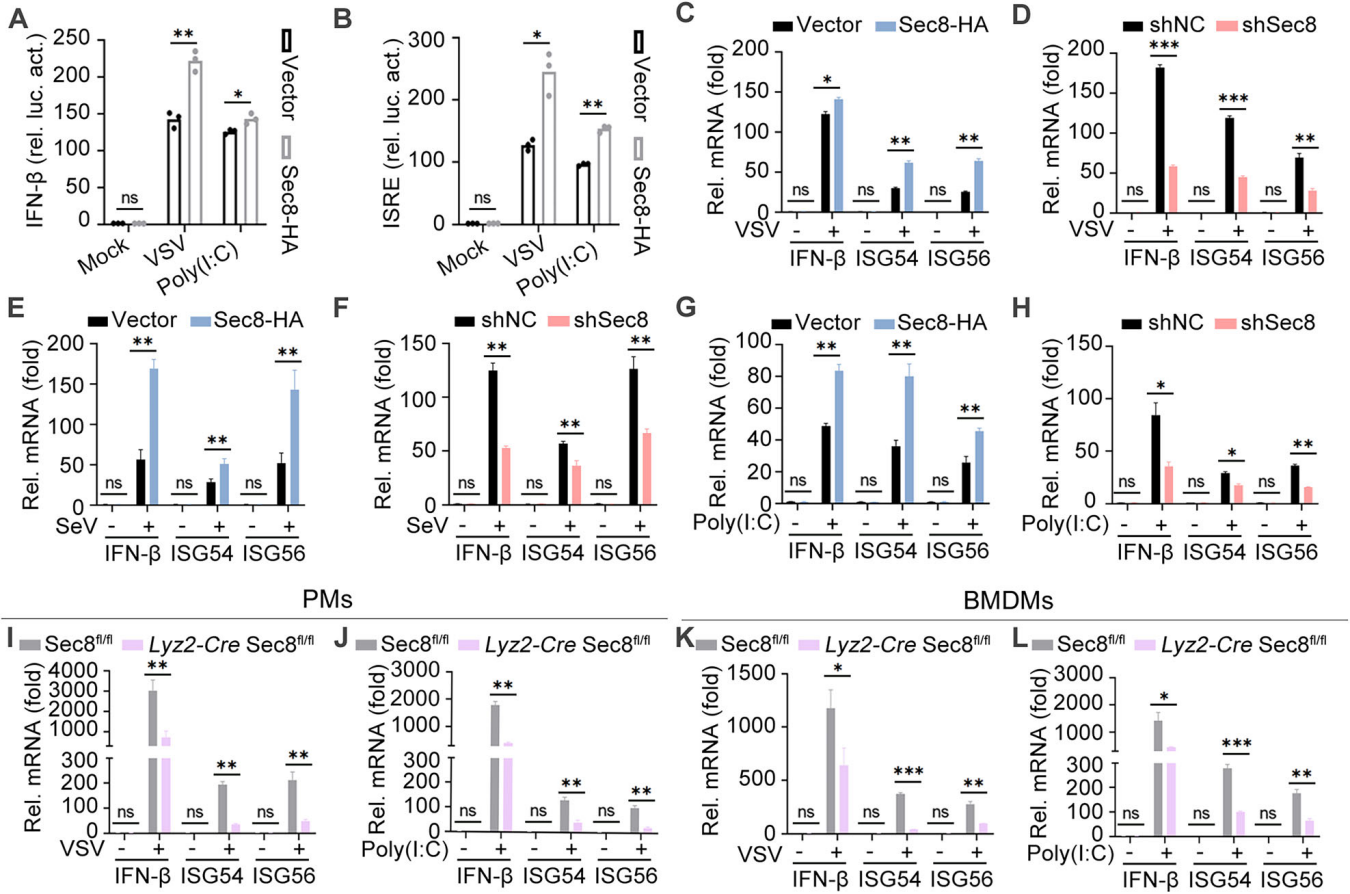
Sec8 enhances the RNA virus-induced IFN-I signaling response in both passaged and primary cells. **A**, **B** Luciferase reporter assays were performed on HEK-293T cells transfected with the indicated plasmids for 24 h and then infected with VSV (MOI = 0.1) or transfected with LWM poly(I:C) (10 μg/mL) for 12 h. The “Mock” group in our experiments refers to cells treated with serum-free DMEM as a control. **C–H** qPCR analysis of the specific genes’ mRNA expression in Sec8-overexpressing or Sec8-silencing HeLa cells infected with VSV (**C**, **D**) or SeV (**E**, **F**); transfected with LWM poly(I:C) (**G**, **H**) for 12 h. qPCR analysis of the specific genes’ mRNA expression in PMs and BMDMs from Sec8^fl/fl^ and *Lyz2-Cre* Sec8^fl/fl^ mice upon VSV infection (**I**, **K**) or LWM poly(I:C) transfection (**J,** **L**) for 12 h. Data are represented as means with standard deviation (SD) derived from three independent experiments. Significance differences were determined by two-way ANOVA, with significance levels denoted as follows: ns not significant; *, *P* < 0.05; **, *P* < 0.01; ***, *P* < 0.001.

To investigate the effect of Sec8 on RNA virus-mediated IFN-I responses, we utilized interferon beta (IFN-β) and IFN-stimulated response element (ISRE) luciferase reporter to assess the activation of the IFN-I signaling response after VSV infection or the viral RNA mimic LWM poly(I:C) transfection. As shown in Fig. 1A, B, overexpressing Sec8 notably promoted IFN-β and ISRE luciferase activities. Next, HeLa cells were transfected with empty vector and Sec8-HA, and we found that overexpressing Sec8 had significantly higher mRNA levels of IFN-β, ISG54, and ISG56 produced after VSV infection, SeV infection, and LWM poly(I:C) transfection compared to control (Fig. 1C, E, G). To further determine the role of Sec8 in the IFN-I signaling response, we constructed Sec8 knockdown HeLa cell lines with stably expressing Sec8-specific shRNA (shSec8) in HeLa cells utilizing lentiviral shRNA technology. Sec8-silencing reduced the mRNA levels of IFN-β, ISG54, and ISG56 in response to VSV infection, SeV infection, or LWM poly(I:C) transfection (Fig. 1D, F, H). The above results suggest that Sec8 promotes RNA virus-triggered type I interferon signaling responses in passaged cells.

Deleting the Sec8 gene is lethal in both mice and Drosophila [31]. In mice, the absence of Sec8 renders mutant embryos viable only to the primitive streak stage, leading to early embryonic mortality [32]. Therefore, we generated the Sec8^fl/+^ mice by CRISPR Cas9-mediated genome editing. Subsequently, we extended our study to examine the effect of Sec8 on the IFN-I signaling response in primary macrophages. Then, peritoneal macrophages (PMs) and bone marrow-derived macrophages (BMDMs) were isolated from Sec8^fl/fl^ and *Lyz2-Cre* Sec8^fl/fl^ mice and treated with VSV or corresponding viral analog LWM poly(I:C). The results indicated that the mRNA levels of IFN-β, ISG54, and ISG56 in PMs from *Lyz2-Cre* Sec8^fl/fl^ mice were significantly diminished compared to those in Sec8^fl/fl^ mice following VSV infection or LWM poly(I:C) transfection (Fig. 1I, J). Similar results were also obtained in BMDMs (Fig. 1K, L). Taken together, these data demonstrate that Sec8 positively regulates RNA virus-induced IFN-I signaling responses in passaged cells and primary macrophages.

### Sec8 promotes IFN-I signaling by upregulating RIG-I

**Fig. 2:**
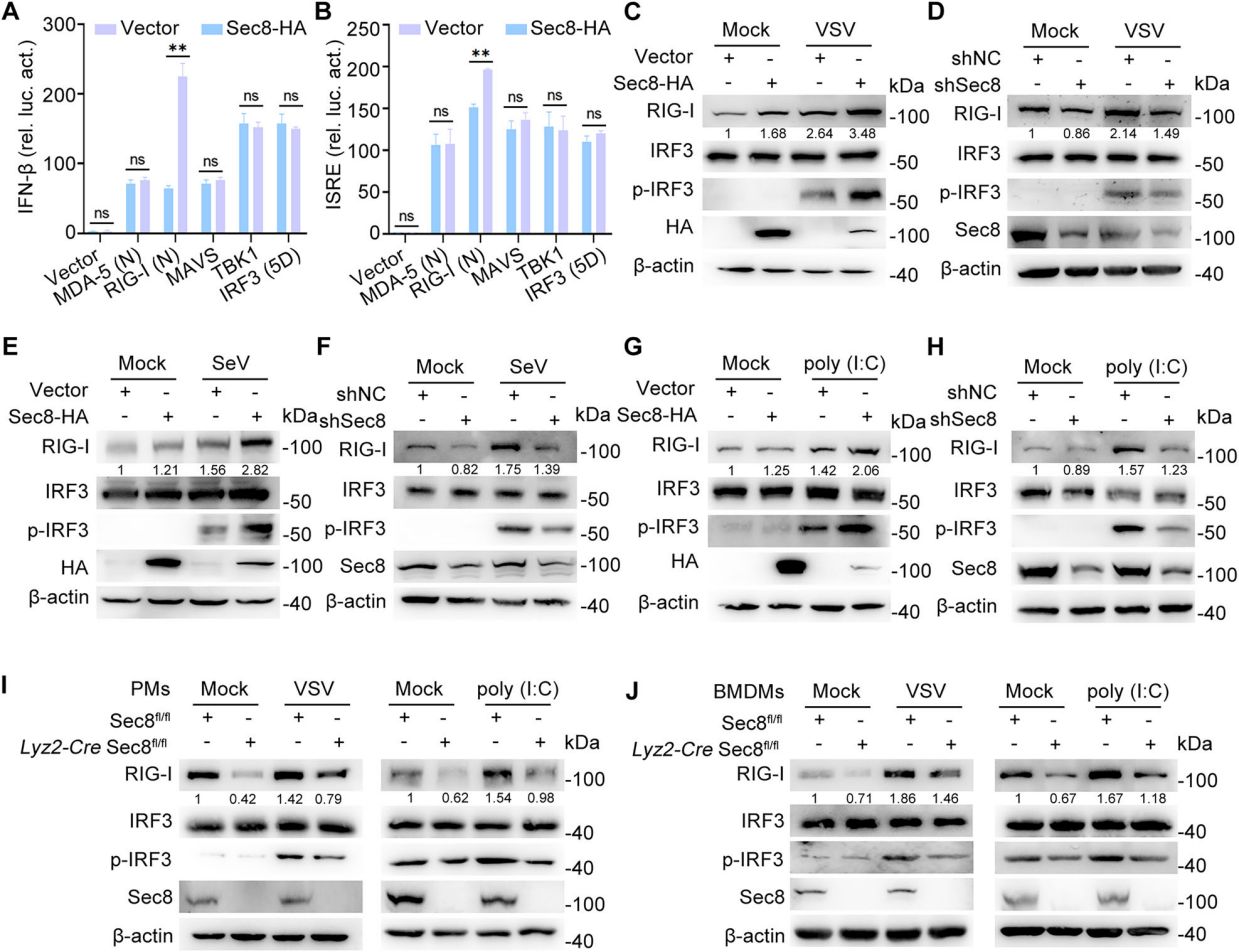
Sec8 facilitates IFN-I signaling response by stabilizing RIG-I. **A**, **B** HEK-293T cells were transfected with the indicated plasmids for 24 h. Subsequently, cells were subjected to luciferase reporter assays. Data are expressed as means with SD from three independent experiments. Significance differences were determined by two-way ANOVA, with significance levels denoted as follows: ns, not significant; **, *P* < 0.01. **C–H** Immunoblot analysis was performed to detect the expression of specified proteins in Sec8-overexpressing and Sec8-silencing HeLa cells following VSV infection (**C**, **D**), SeV infection (**E**, **F**), or LWM poly(I:C) transfection (**G**, **H**) for 12 h. **I**, **J** Immunoblot analyses of indicated protein expression in PMs and BMDMs from Sec8^fl/fl^ and *Lyz2-Cre* Sec8^fl/fl^ following VSV infection or LWM poly(I:C) transfection for 12 h.

To investigate the regulatory mechanism of Sec8 in IFN-I signaling response during RNA viral infection, we conducted a dual-luciferase reporter assay to measure the activation of the IFN-β promoter and ISRE promoter in response to the RLR signaling cascade in the Sec8-overexpressing or control groups. Our results revealed that overexpressing Sec8 enhanced the activation of IFN-β and ISRE promoters triggered by RIG-I but not MAD-5, MAVS, TBK1, or IRF3-5D (a constitutively activated form of IRF3) (Fig. 2A, B). In addition, we found that overexpressing or silencing Sec8 did not affect the mRNA expression of IFN-β via MDA-5, TLR3, and TLR4 respective stimuli (Fig. S1A, S1B). These results suggest that Sec8 may act on RIG-I during RNA virus infection. Furthermore, overexpression of Sec8 resulted in a significant increase in the expression of endogenous RIG-I during VSV infection, SeV infection, or following LWM poly(I:C) transfection (Fig. 2C, E, G). Conversely, Sec8-silencing decreased endogenous protein levels of RIG-I (Fig. 2D, F, H). Notably, overexpression of Sec8 facilitated IRF3 phosphorylation triggered by VSV infection, SeV infection, or LWM poly(I:C) transfection (Fig. 2C, E, G), while silencing Sec8 elicited the opposite results (Fig. 2D, F, H), further indicating that Sec8 enhances RNA virus-induced IFN-I signaling response. Similarly, total protein levels of RIG-I, as well as the phosphorylated forms of IRF3, were remarkably diminished in PMs and BMDMs from *Lyz2-Cre* Sec8^fl/fl^ compared to control Sec8^fl/fl^ during VSV infection or following LWM poly(I:C) transfection (Fig. 2I, J). Collectively, these data suggest that Sec8 functions as a positive regulator of IFN-I signaling responses by upregulating RIG-I upon RNA viral infection.

### Sec8 blocks STUB1-mediated ubiquitination of RIG-I at Lys190 to prevent its proteasomal degradation

**Fig. 3:**
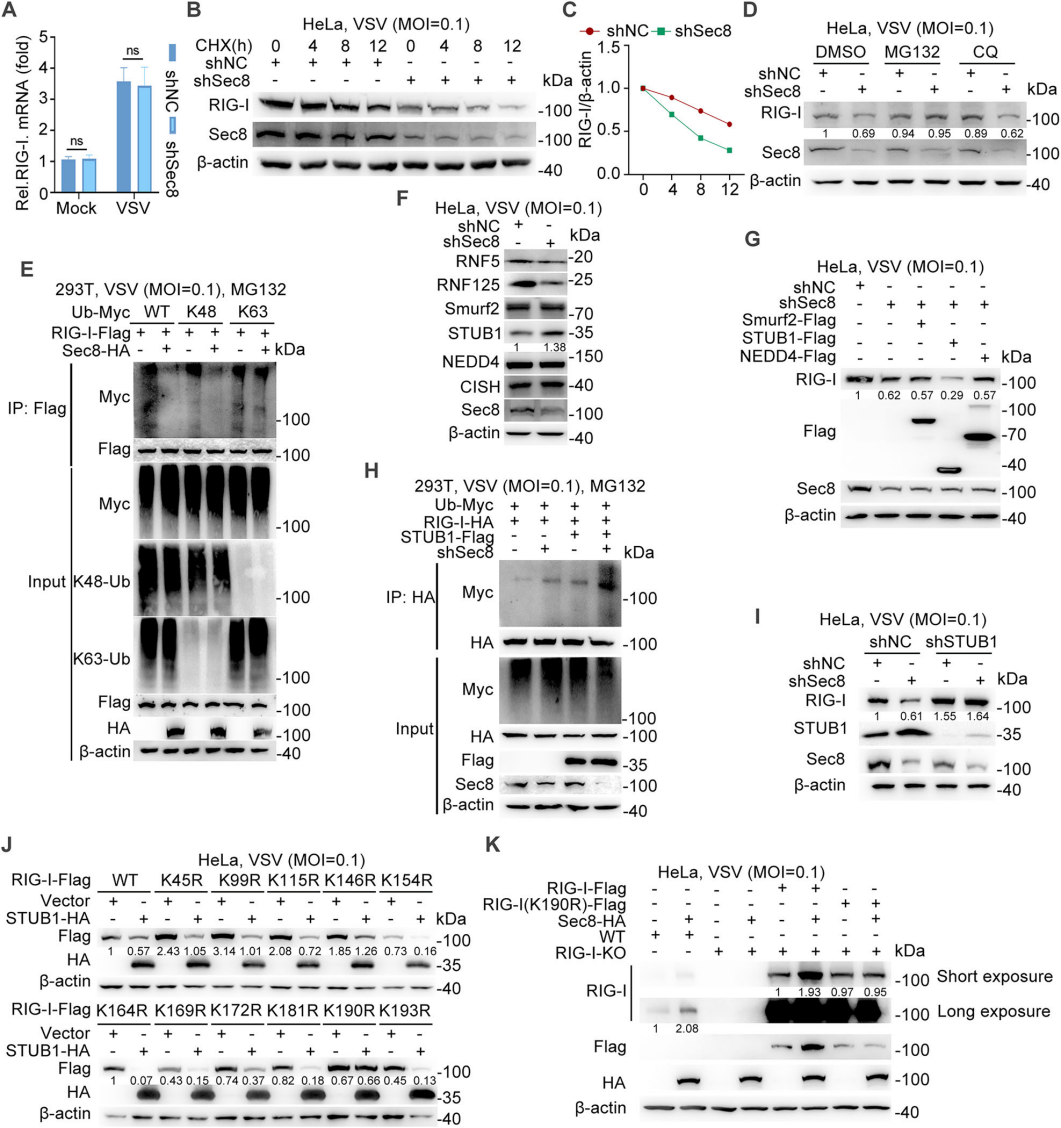
Sec8 inhibits STUB1-mediated ubiquitin-proteasome degradation of RIG-I at K190. **A** qPCR analysis of RIG-I mRNA expression in Sec8-silencing HeLa cells infected with VSV for 12 h. Significance differences were determined by two-way ANOVA, with significance levels denoted as follows: ns, not significant. **B** Immunoblot analysis of specified proteins in VSV-infected Sec8-silencing HeLa cells, followed by treatment with cycloheximide (CHX) (100 μg/mL) for the indicated time intervals. **C** Quantification of RIG-I protein expression in (**B**) by band intensity using ImageJ software. **D** Immunoblot analysis of specified proteins in control or Sec8-silencing HeLa cells was performed after they were treated with DMSO, MG132 (10 μM), or CQ (20 μM) and infected with VSV for 12 h. **E** Evaluation of various lysine ubiquitination types (wild type, K48, and K63) on RIG-I by Sec8 was performed by co-transfecting HEK-293T cells with relevant plasmids, followed by MG132 treatment and VSV infection (MOI = 0.1) for 12 h. **F** Immunoblot analysis of specified proteins in VSV-infected Sec8-silencing HeLa cells. **G** Immunoblot analysis of RIG-I expression in Sec8-silencing HeLa cells transfected with the indicated E3 ligase plasmids. **H** The ubiquitination of RIG-I was evaluated through co-transfection of HEK-293T cells with the indicated plasmids for co-immunoprecipitation assays. **I** Immunoblot analysis of RIG-I protein level in Sec8-silencing HeLa cells transfected with shSTUB1 or negative control (shNC). **J** Immunoblot analysis of wild-type RIG-I and various RIG-I mutants expressed in HeLa cells cotransfected with wild-type RIG-I or RIG-I mutated at lysine sites in the presence or absence of STUB1. **K** Immunoblot analysis of RIG-I expression with reintroduced wild-type RIG-I or RIG-I-K190R mutant with empty vector or Sec8-HA into RIG-I-KO HeLa cells upon VSV infection.

To elucidate the mechanism by which Sec8 upregulates RIG-I expression upon RNA viral infection, we assessed the impact of Sec8 on RIG-I mRNA level during VSV infection. The results showed that the knockdown of Sec8 did not affect the mRNA expression of RIG-I in response to VSV infection (Fig. 3A). In addition, silencing of Sec8 accelerated the degradation of RIG-I after treatment with cycloheximide (CHX) during VSV infection (Fig. 3B, C), indicating that Sec8 has no effect on the RIG-I mRNA level during virus infection. Given that RIG-I degradation occurs primarily through the ubiquitin-proteasome and autophagy-lysosome pathways, we subsequently examined changes in silencing Sec8-mediated RIG-I degradation in response to the ubiquitin-proteasome inhibitor MG132 and the autophagy-lysosome pathway inhibitor chloroquine (CQ). As shown in Fig. 3D, the downregulation of RIG-I following Sec8 silencing could be prevented by MG132 but not CQ, indicating RIG-I degradation caused by Sec8 silencing occurs mainly through the ubiquitin-proteasomal pathway.

Next, we investigated the involvement of Sec8 in RIG-I ubiquitination during RNA virus infection. Under VSV infection, Sec8 inhibited wild-type and K48-linked polyubiquitination of RIG-I but not K6, K11, K27, K29, K33, or K63-linked polyubiquitination (Figs. 3E and S2A). In addition, we observed that Sec8 inhibited RIG-I ubiquitination in the presence of wild-type ubiquitin, K63R ubiquitin (which cannot form K63-linked chains), but did not exhibit this effect with K48R ubiquitin (which cannot form K48-linked chains) (Fig. S2B). The above results indicate that Sec8 specifically abrogates K48-linked ubiquitination of RIG-I. E3 ubiquitin ligases function as determinants of target protein-specific recognition during protein degradation [33]. Therefore, we need to find the E3 ubiquitin ligase that mediates the ubiquitin-proteasome pathway degradation of RIG-I that occurs in silencing Sec8 VSV-infected cells. Next, we predicted E3 ubiquitin ligases through the ubibrowser (http://ubibrowser.bio-it.cn/) that may have degrading effects on RIG-I, including RNF5, RNF125, Smurf2, STUB1, NEDD4, and CISH. We examined the protein levels of these E3 ubiquitin ligases in silenced Sec8 cells infected with VSV. The results show that silencing Sec8 promotes the protein levels of STUB1 but not other E3 ubiquitin ligase proteins (Fig. 3F). Furthermore, Sec8 exhibited dose-dependent inhibition of STUB1 protein levels (Fig. S2C). Next, we transfected a part of the E3 ubiquitin ligases-plasmids in Sec8 knockdown cell lines and found that the protein expression of RIG-I was further down-regulated after overexpression of STUB1 but not Smurf2 and NEDD4 (Fig. 3G). Consequently, we speculated that STUB1 might be an E3 ubiquitin ligase targeting RIG-I that is regulated by Sec8. Silencing Sec8 and overexpressing STUB1 produced a markedly higher rate of RIG-I ubiquitination than silencing Sec8 or overexpressing STUB1 individually (Fig. 3H). Furthermore, the degradation of RIG-I in the silencing Sec8 cell lines was eliminated following STUB1 knockdown during VSV infection (Fig. 3I), whereas silencing Smurf2 did not produce this effect (Fig. S2D), suggesting that STUB1 plays an important role in the Sec8-regulated RIG-I expression. It is reported that STUB1 degraded RIG-I by ubiquitinating the 2CARD structural domain of RIG-I [34]. To investigate the specific lysine residues in RIG-I ubiquitination and degradation by STUB1, we engineered a series of RIG-I mutants where lysine residues were substituted with arginine (K/R mutants) and co-transfected these constructs with STUB1 during virus infection, respectively. The result demonstrated that STUB1 was effective in downregulating the expression of RIG-I mutants, except for the K190R mutant (Fig. 3J). This observation suggests that lysine 190 is critical for STUB1-induced ubiquitination of RIG-I. Furthermore, we found that overexpression of STUB1 or silencing of Sec8 significantly promoted K48-linked ubiquitination of wild-type RIG-I. This effect was further augmented by the combined overexpression of STUB1 and silencing of Sec8. However, this effect was not observed with the RIG-I-K190R mutant (Fig. S2E), demonstrating that Sec8 inhibits STUB1-mediated K48-linked ubiquitination of RIG-I specifically at K190. Additionally, we generated a RIG-I-knockout (RIG-I-KO) HeLa cell line utilizing the CRISPR/Cas9 system. Upon introduction with either RIG-I or RIG-I-K190R mutant in RIG-I-KO cell lines, we observed that overexpression of Sec8 promoted the expression of wild-type RIG-I but did not affect the expression of the K190R mutant (Fig. 3K). All in all, our findings indicate that STUB1 degrades RIG-I by promoting the K48-linked ubiquitination of RIG-I at lys190, while Sec8 inhibits this process.

### Sec8 represses mRNA expression of STUB1 by decreasing the protein of p53

**Fig. 4:**
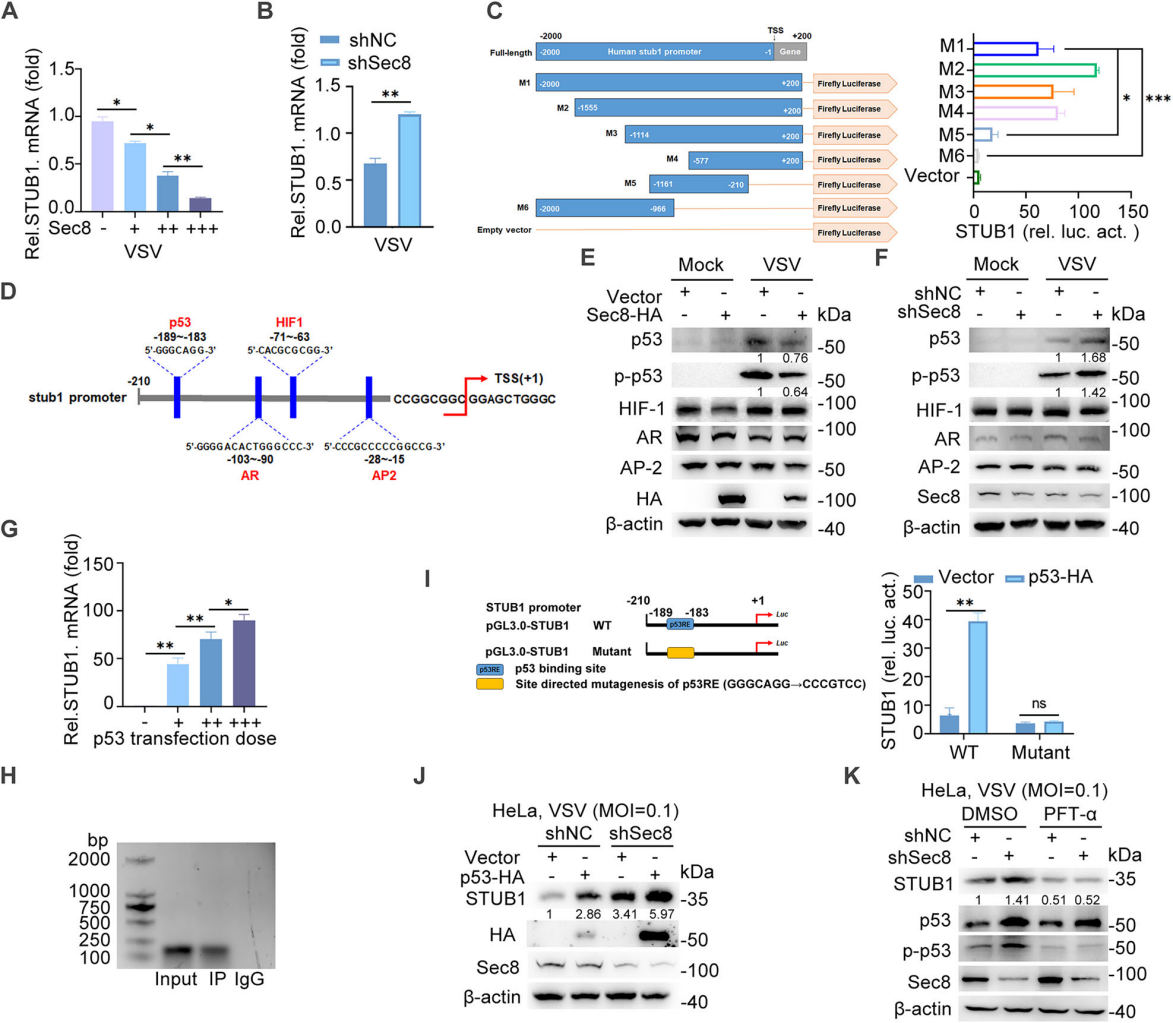
Sec8 promotes the IFN-I signaling response by inhibiting the p53-mediated transcription of STUB1. **A** qPCR analysis of STUB1 expression following transfection with varying concentrations of Sec8-HA (0.6, 1.2, or 1.8 μg/mL) or empty plasmid in HeLa cells infected with VSV. Significance differences were determined by one-way ANOVA in (**A**–**C**, and **G**), two-way ANOVA in I, with significance levels denoted as follows: ns, not significant; *, *P* < 0.05; **, *P* < 0.01; ***, *P* < 0.001. **B** qPCR analysis of STUB1 in Sec8-silencing HeLa cells infected with VSV. **C** HEK-293T cells were co-transfected with distinct truncated STUB1 promoter constructs (−2000 to +200, M1 to M6) alongside Renilla luciferase reporter vector (pRL-TK-luc) for dual luciferase activity analysis. **D** Predicted transcription factors obtained that may target the STUB1-210 to +1 promoter region. **E**, **F** Immunoblot analysis was performed for the specified proteins in Sec8-overexpressing and Sec8-silencing HeLa cells infected with VSV. **G** qPCR analysis of STUB1 following transfection with varying concentrations of p53-HA (0.6, 1.2, or 1.8 μg) or empty plasmid for 24 h in HeLa cells infected with VSV. **H** ChIP was performed in HEK-293T cells with p53 antibody. The interaction between the p53 protein and the STUB1 promoter region was analyzed by PCR with STUB1-designated primers. **I** A schematic representation of STUB1 promoter mutations. Luciferase reporter assays were conducted with HEK-293T cells transfected for 24 h with STUB1-WT (−210 to +1) or STUB1-mutant (−210 to +1) together with p53-HA or control plasmid for 24 h. Data are shown as means with SD from three independent experiments. Statistical significance is marked as: ns, not significant; *, *P* < 0.05; **, *P* < 0.01; ***, *P* < 0.001. **J** Immunoblot analysis of STUB1 in Sec8-silencing HeLa cells transfected with empty vector or p53-HA infected with VSV (MOI = 0.1) for 12 h. **K** Immunoblot analysis of STUB1 in control or Sec8-silencing HeLa cells treated with DMSO or PFT-α (20 μM) and infected with VSV (MOI = 0.1) for 12 h.

Notably, we observed a marked increase in the expression of STUB1 following silencing of Sec8 (Fig. 3F). Therefore, we further explored how Sec8 affects STUB1 expression. The mRNA level of STUB1 was determined. As shown in Fig. 4A, overexpression of Sec8 exerted a dose-dependent inhibitory effect on the mRNA level of STUB1. In contrast, silencing Sec8 resulted in an elevation of STUB1 mRNA levels (Fig. 4B). These results suggest that Sec8 represses STUB1 expression at the transcriptional level. Since Sec8 does not function as a transcription factor, we posited that Sec8 may inhibit STUB1 mRNA expression by modulating a specific transcription factor. Subsequently, we constructed a series of truncations (designated M1–M6) of the STUB1 promoter sequence luciferase vector (pGL_3.0_-basic) to assess their ability to activate luciferase expression in HEK-293T cells. Notably, the M5 and M6 constructs exhibited lower luciferase activity, while the other truncations yielded high levels of activation (Fig. 4C). This observation led us to infer that the region of −210 to +1 bp encompasses the core promoter of STUB1. Furthermore, we utilized the JASPAR database (https://jaspar.elixir.no/) to predict transcription factors that may target this promoter region (Fig. 4D). Subsequently, the transcription factors were screened through overexpression or silencing of Sec8. The results showed that overexpression of Sec8 suppressed the expression of total p53 protein and its phosphorylation levels, while knockdown of Sec8 had the opposite effect (Fig. 4E, F). Importantly, the phosphorylation of p53 at Ser33 is closely associated with its transcriptional activity in cellular processes [35–37]. The above results indicate that p53 may play a role in regulating STUB1 mediated by Sec8. Additionally, we observed that p53 increased the STUB1 mRNA level in a dose-dependent way (Fig. 4G). ChIP assays confirmed that p53 bound to the −210 to +1 region of the STUB1 gene (Fig. 4H). In addition, p53 protein accumulated at specific regions of the STUB1 promoter in the control group, with this enrichment significantly enhanced upon Sec8 knockdown (Fig. S3A). The result directly demonstrates that p53 binds to the STUB1 promoter and that this interaction is inhibited by Sec8.

By mutating all predicted p53-binding sites within the STUB1 promoter, dual-luciferase reporter assays revealed that p53 no longer enhanced the activity of the mutant STUB1 promoter in comparison to the wild-type promoter (−210 to +1) (Fig. 4I). Moreover, overexpression of p53 resulted in a further increase in STUB1 expression relative to the effects observed with the silencing of Sec8 alone (Fig. 4J). In contrast, the increase in protein expression of STUB1 induced by silencing Sec8 was abolished after pifithrin-α (PFT-α, an inhibitor of p53 phosphorylation) treatment, which suppresses p53-dependent gene transcriptional activity but did not affect p53 expression at the protein level (Fig. 4K). Importantly, we further revealed that knocking down p53 in Sec8-silenced cell lines led to a significant reduction in phospho-p53 (Ser33) levels and abolished the suppression of STUB1 expression (Fig. S3B). k48 data suggest that Sec8 inhibition of STUB1 mRNA level is dependent on the expression of p53 and its role as a transcription factor. In conclusion, these findings demonstrate that Sec8 promotes the RLR signaling pathway by repressing RIG-I degradation via inhibiting the p53-mediated transcription of STUB1.

### Sec8 stabilizes RIG-I by competitively binding the 2CARD domain of RIG-I with STUB1

**Fig. 5:**
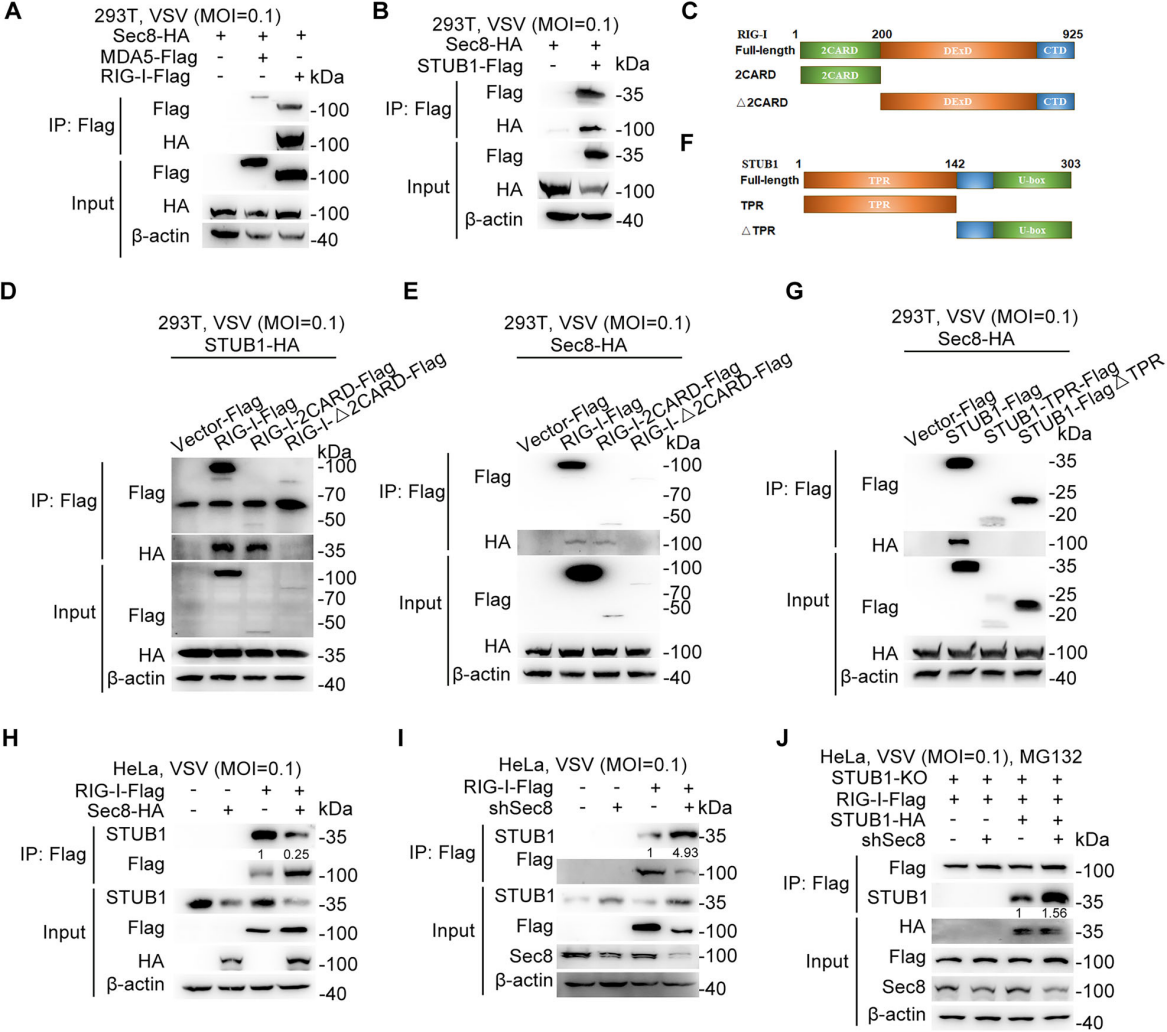
Sec8 competitively binds the 2CARD domain of RIG-I with STUB1. **A** Immunoprecipitation assays investigated the interaction between Sec8 and MDA5 or RIG-I. **B** Immunoprecipitation assays investigated the interaction between Sec8 and STUB1. **C** Models for the RIG-I truncated mutant. **D**, **E** Immunoprecipitation and immunoblotting analysis of the interaction of STUB1 or Sec8 with the RIG-I truncated mutant. **F** Models for the STUB1 truncated mutant. **G** Immunoprecipitation and immunoblotting analysis of the interaction of Sec8 with the STUB1 truncated mutant. **H**, **I** Immunoprecipitation analysis of the interaction between STUB1 and RIG-I in Sec8-overexpressing or Sec8-silencing HeLa cells infected with VSV for 12 h. **J** Immunoprecipitation analysis of the interaction between STUB1 and RIG-I in STUB1-KO cells infected with VSV for 12 h with MG132 treatment.

Immunoprecipitation results showed that Sec8 interacted with RIG-I but not MDA5 (Fig. 5A). Meanwhile, we also found that Sec8 interacted with STUB1 (Fig. 5B). In addition, we constructed N-terminal truncates of RIG-I containing the 2CARD structural domain and truncates lacking the 2CARD structural domain (Fig. 5C). The immunoprecipitations demonstrated that both Sec8 and STUB1 interacted only with the 2CARD structural domain of RIG-I (Fig. 5D, E). This result implies that the 2CARD domain is important for the interaction between RIG-I with Sec8 or STUB1. Moreover, we constructed a truncated form of STUB1 (Fig. 5F). We found that Sec8 can only interact with intact STUB1 (Fig. 5G). All these results indicate that Sec8 and STUB1 can competitively bind the 2CARD domain of RIG-I. To confirm whether Sec8 affects the interaction between RIG-I and STUB1, we examined their association when Sec8 was overexpressed or knocked down. Additionally, immunoblot results revealed that Sec8 inhibited the interaction between STUB1 and RIG-I (Fig. 5H, I). However, it cannot be excluded in this process that the attenuated interaction of STUB1 with RIG-I is due to the effect of Sec8 on the mRNA expression of STUB1. In order to exclude this effect, we constructed STUB1 knockout cell lines (STUB1-KO) and re-introduced STUB1 in knockout STUB1 cells, thus ensuring consistent expression of STUB1 at the protein level (Fig. S4A). We found that silencing Sec8 still could promote the interaction of STUB1 with RIG-I (Fig. 5J). Besides, in STUB1-knockout cells reintroduced with STUB1, we assessed Sec8’s impact on the interaction between endogenous RIG-I and STUB1. The results showed that increasing doses of Sec8 gradually reduced the RIG-I-STUB1 interaction (Fig. S4B). In the STUB1-reintroduced cells, we also examined the effect of Sec8 knockdown on the interaction between STUB1 with full-length RIG-I or its domain mutants (RIG-I-2CARD and RIG-I-Δ2CARD). The results revealed that Sec8 knockdown enhanced the interaction between STUB1 with both full-length RIG-I and the RIG-I-2CARD mutant, but no interaction was detected between STUB1 with the RIG-I-Δ2CARD mutant (Fig. S4C). Collectively, the above results suggest that Sec8 inhibits the STUB1-mediated degradation of RIG-I through competitive binding of the 2CARD structural domain of RIG-I with STUB1.

### Sec8 suppresses RNA virus replication by inhibiting the p53-STUB1-RIG-I axis in vitro

**Fig. 6:**
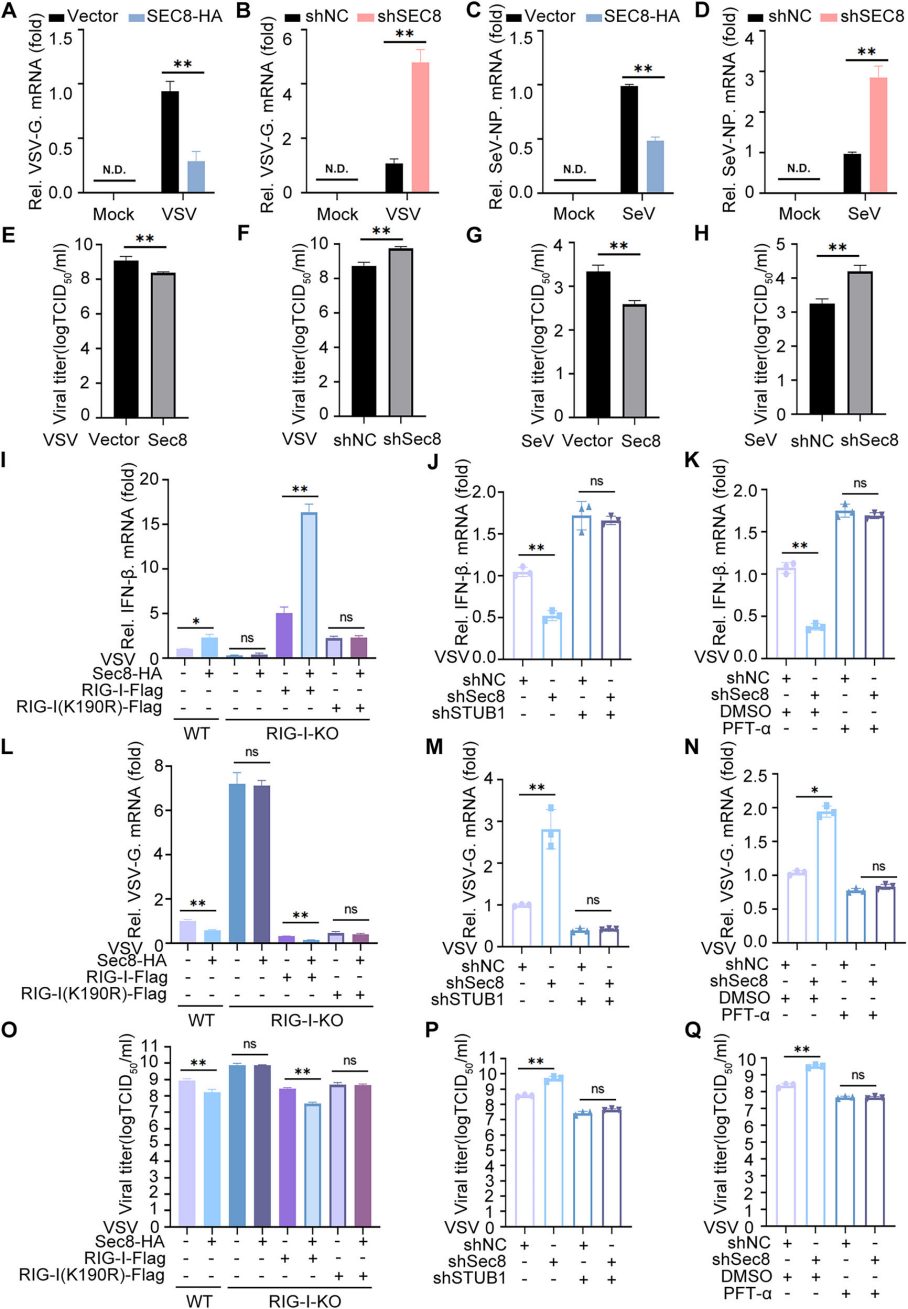
Sec8 suppresses RNA viral replication by negatively regulating the p53-STUB1-RIG-I axis. **A–D** qPCR was conducted to assess the mRNA levels of VSV-G or SeV-NP in Sec8-overexpressing or Sec8-silencing HeLa cells during VSV infection (MOI = 0.1) or SeV infection (MOI = 0.1), which lasted for 12 h. **E–H** TCID_50_ analysis of viral titers after VSV or SeV infection for 12 h in Sec8-overexpressing or Sec8-silencing cells. **I**, **L**, **O** RIG-I-KO HeLa cells were similarly transfected with Sec8 and either wild-type RIG-I or RIG-I-K190R mutant for 24 h, followed by VSV infection (MOI = 0.1) for 12 h, followed by qPCR and TCID_50_. **J, K, M, N, P, Q** Analyses of qPCR or TCID_50_ were conducted after VSV (MOI = 0.1) infection in Sec8-silenced HeLa cells that were transfected with STUB1 shRNA or treated with PFT-α. Data are presented as means with SD from three independent experiments. Significance differences were determined by two-way ANOVA in (**A**–**D**), one-way ANOVA in (**E**–**Q**), with significance levels denoted as follows: ns not significant; *, *P* < 0.05; **, *P* < 0.01.

Next, we investigated whether Sec8 plays a regulatory role in RNA virus replication. The qPCR assays demonstrated that Sec8 overexpression decreased the mRNA levels of VSV-G and SeV-NP (Fig. 6A, C), whereas silencing Sec8 yielded the opposite effects (Fig. 6B, D). Correspondingly, Sec8 overexpression reduced VSV and SeV titers (Fig. 6E, G), while silencing Sec8 increased these viral titers (Fig. 6F, H). These results indicate that Sec8 suppresses the replication of RNA viruses. To further investigate whether Sec8 regulates the IFN-I signaling response and RNA viral replication through RIG-I, we examined the mRNA level of IFN-β after reintroducing wild-type RIG-I and the RIG-I-K190R mutant in RIG-I-knockout (RIG-I-KO) cells. As shown in Fig. 6I, Sec8 overexpression did not enhance IFN-β mRNA level in RIG-I-KO cells relative to control cells. However, when wild-type RIG-I was reintroduced, Sec8 increased the IFN-β mRNA level, but this effect was not seen with the RIG-I-K190R mutant. Additionally, Sec8 overexpression did not decrease VSV-G mRNA levels in RIG-I-KO cells compared to controls. In contrast, reintroducing wild-type RIG-I in RIG-I-KO cells resulted in a significant reduction in VSV-G mRNA levels following Sec8 overexpression, which was absent in the presence of the RIG-I-K190R mutant (Fig. 6L). The 50% Tissue Culture Infective Dose (TCID_50_) result was in keeping with the mRNA level for VSV-G (Fig. 6O). These findings suggest that Sec8 inhibits RNA virus replication by stabilizing RIG-I and enhancing IFN-I signaling. Notably, silencing STUB1 or treating with PFT-α restored the IFN-β mRNA levels that decreased by Sec8 silencing, as compared to the control group (Fig. 6J, K). Furthermore, silencing STUB1 or treating with PFT-α mitigated the increased mRNA level of VSV-G and viral titer of VSV induced by Sec8 silencing (Fig. 6M, N, P, Q). In summary, our results indicate that Sec8 plays a crucial role in facilitating the IFN-I signaling response and inhibiting RNA virus replication by suppressing the p53-STUB1-RIG-I axis.

### The absence of Sec8 is more favorable for RNA viral replication in vivo

**Fig. 7:**
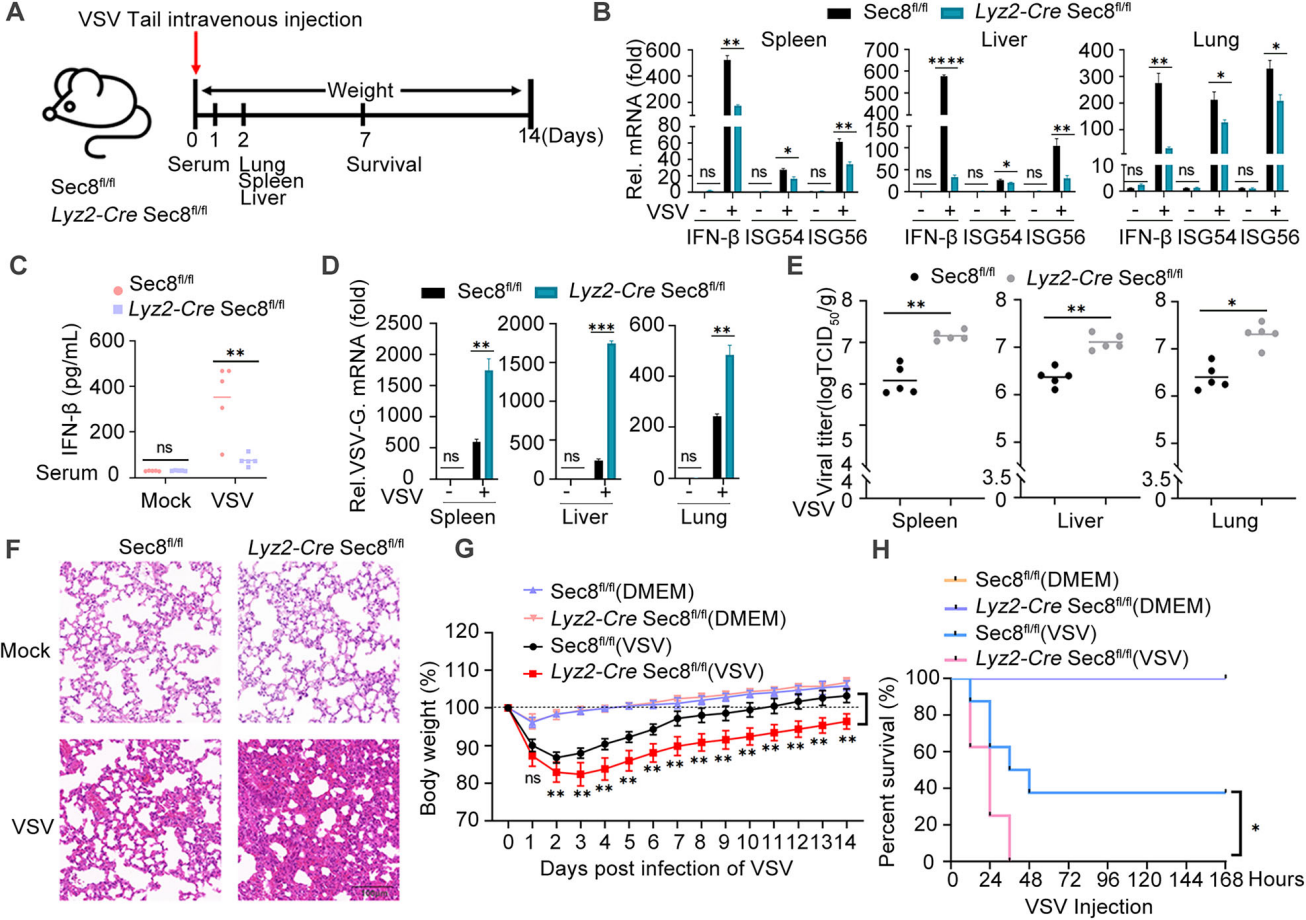
Sec8 deficiency enhances RNA virus replication in vivo. **A** Schematic representation of the VSV infection and subsequent analysis. Following a 48-h infection via tail vein injection of VSV at a dose of 2 × 10^7^ plaque-forming units (PFU) per mouse, Sec8^fl/fl^ and *Lyz2-Cre* Sec8^fl/fl^ mice (*n* = 5 mice per group) underwent analysis wherein and (**B**) qPCR analysis of the specified genes in the spleen, liver, and lungs; **D** qPCR analysis of the mRNA of VSV-G; **E** TCID_50_ analysis of viral titer. **C** ELISA analysis of IFN-β levels in serum of Sec8^fl/fl^ and *Lyz2-Cre* Sec8^fl/fl^ mice (*n* = 5 mice per group) following 24-h VSV infection via tail vein injection. **F** Histopathological evaluation through hematoxylin and eosin staining of lung tissue sections derived from Sec8^fl/fl^ and *Lyz2-Cre* Sec8^fl/fl^ mice with VSV infection. Scale bars denote 100 µm. **G**, **H** Body weight and survival (Kaplan‒Meier curves) were monitored in Sec8^fl/fl^ and *Lyz2-Cre* Sec8^fl/fl^ mice (*n* = 8 mice per group) injected by tail vein with VSV (2 × 10^7^ PFU per mouse and 1 × 10^8^ PFU per mouse). The mean and SD from three independent experiments are shown. Significance differences were determined by two-way ANOVA in (**B**–**D**, and **G**), Student’s *t* test in (**E**), and the log-rank (Mantel-Cox) test in (**H**), with significance levels denoted as follows: ns not significant; *, *P* < 0.05, **, *P* < 0.01, and ***, *P* < 0.001.

To further assess the physiological significance of our work, we investigated various changes in antiviral host defense in wild-type or Sec8-deficient mice following VSV infection (Fig. 7A). The mRNA levels of IFN-β, ISG54, and ISG56 in *Lyz2-Cre* Sec8^fl/fl^ mice in the spleen, liver, and lungs were significantly lower than Sec8^fl/fl^ (Fig. 7B). Correspondingly, IFN-β protein in serum from *Lyz2-Cre* Sec8^fl/fl^ mice was also markedly diminished compared to Sec8^fl/fl^ mice (Fig. 7C). These results suggest that deletion of Sec8 significantly inhibits type I IFN signaling responses in vivo. Furthermore, the VSV-G mRNA level and viral titer were markedly elevated in *Lyz2-Cre* Sec8^fl/fl^ mice compared to control mice (Fig. 7D, E). Moreover, by observing the pathological damage of alveolar wall thickening after VSV infection in both types of mice, the results showed that *Lyz2-Cre* Sec8^fl/fl^ mice showed alveolar congestion and edema, and alveolar wall thickening was more pronounced (Fig. 7F). Body weight was monitored within 14 days after infection of each mouse with 2 × 10^7^ PFU of VSV, and the number of mice surviving was counted within 7 days after infection of each mouse with 1 × 10^8^ PFU of VSV. All mice showed a decrease in body weight on the first day after viral infection, with the decline persisting into the second day. Sec8^fl/fl^ grew faster from day 3 to day 14 (Fig. 7G). In addition, the survival rate of *Lyz2-Cre* Sec8^fl/fl^ mice was significantly lower than Sec8^fl/fl^ mice after infection with VSV (Fig. 7H). These findings demonstrated that Sec8-deficient mice exhibited a poorer IFN-I response to RNA virus infection, thereby enhancing viral replication, promoting morbidity, and reducing survival.

## Conclusion

**Fig. 8:**
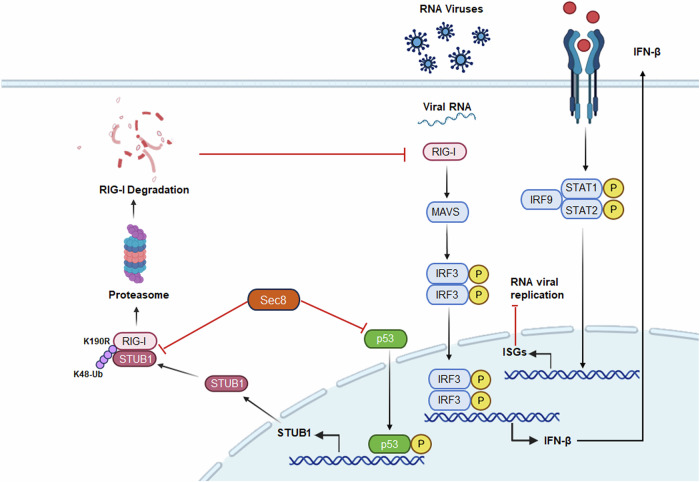
The working model of Sec8 negatively regulates RNA viral replication by inhibiting the p53-STUB1-RIG-I axis. Upon RNA virus infection, Sec8 not only inhibits STUB1 transcription by suppressing p53 protein expression but also competitively binds RIG-I with STUB1, thereby preventing RIG-I from ubiquitin-proteasome degradation, which promotes the antiviral innate immunity and depresses the RNA virus replication.

RIG-I is a cytosolic pattern recognition receptor that activates IFN-I signaling by detecting viral RNA [4, 38]. Deficiency of RIG-I results in severely reduced host interferon production and high susceptibility to RNA viruses, including paramyxoviruses, influenza viruses, and Japanese encephalitis viruses, thus demonstrating that precise regulation of RIG-I is essential for host antiviral responses [39]. Our study identified Sec8 as a specific positive regulator for RIG-I in both passaged and primary cells. Deficiency of Sec8 results in reduced production of type I interferons and ISGs in response to RNA virus infections, leading to weakened innate immune responses, increased viral load, and higher in vivo morbidity (Fig. 8).

Ubiquitination modifications are crucially important for RIG-I degradation. At least three E3 ubiquitin ligases have been reported to be involved in RIG-I degradation due to polyubiquitination. For instance, RNF125 mediates polyubiquitination of the K48 linkage of RIG-I for its degradation via the proteasome, thereby inhibiting RIG-I-induced IFN-I production [2]; TRIM40, an E3 ubiquitin ligase, promotes polyubiquitination of the K27 and K48 linkages of RIG-I and attenuates RLR-triggered signaling [19]. Interestingly, it has been reported that STUB1 is an E3 ubiquitin ligase that can target RIG-I for K48-linked ubiquitination and promote its degradation [6]. However, in our study, we demonstrated for the first time that STUB1 can target the lysine 190 position of RIG-I for ubiquitination and degradation. This discovery enriches the ubiquitination modification site of RIG-I, which has not been previously reported to be ubiquitinated at this site. Furthermore, it is well reported that host genes regulate the transcriptional level of E3 ubiquitin ligases, which subsequently affects the expression of E3 ligase target proteins [40]. Two genes have been demonstrated to be associated with STUB1 transcription, in which IL-1α promotes STUB1 expression through transactivation mediated by the transcription factor ELK1 [41], and XBP1s can act as a transcription factor that binds to the STUB1 promoter to promote STUB1 transcription [42]. P53 is a transcription factor that regulates the transcriptional activity of its target proteins [43, 44]. However, no study has yet demonstrated that p53 is associated with STUB1 transcription. Here, we revealed for the first time that p53 plays an actor as a transcription factor of STUB1 to promote its mRNA expression. In this study, we also discovered that Sec8 depresses the mRNA level of STUB1 transcription by inhibiting p53, thereby preventing RIG-I from degradation. In exploring the transcription factors of STUB1 regulated by Sec8, we found that Sec8 not only down-regulates the protein expression of p53 but also inhibits its phosphorylation at the serine 33 position, thereby impairing its activity as a transcription factor and ultimately inhibiting STUB1 transcription. However, how Sec8-mediated downregulation of the p53 expression needs to be further researched.

In addition, we demonstrated that Sec8 competes with STUB1 for binding to the 2CARD domain of RIG-I and thus inhibits the degradation of RIG-I. Specifically, in STUB1-KO cells, Sec8 still restrained the interaction between STUB1 and RIG-I after excluding the effect of Sec8 on endogenous STUB1 transcript levels. In addition, both Sec8 and STUB1 bind only to the CARD structural domain of RIG-I. In conclusion, Sec8 promotes the IFN-I response at the level of passaged cells, primary macrophages, and mice serum, respectively, through the two mechanisms described above.

In summary, we describe the mechanism by which Sec8 stabilizes RIG-I to promote antiviral innate immunity and inhibit RNA virus replication in vivo and in vitro. Our findings indicate that Sec8 inhibits the mRNA expression of STUB1 and its binding to RIG-I, thereby enhancing the antiviral signaling response by preventing the RIG-I degradation during RNA virus infection. More interestingly, we identified p53 as a new transcription factor of STUB1, which is inhibited by Sec8 in STUB1 transcription. Our study demonstrates for the first time the importance of Sec8 for the inhibition of viral infection and the promotion of host innate immunity.

## Methods and materials

### Cell culture and viruses

The HEK-293T (CRL-11268) and HeLa (CCL-2) cells were obtained from the American Type Culture Collection (ATCC). HEK-293T and HeLa cell lines were maintained in Dulbecco’s modified Eagle’s medium (DMEM; VivaCell, C3110-0500) containing 10% fetal bovine serum (FBS; TransGen Biotech, FS301-02) under a humidified atmosphere of 5% CO_2_ at 37 °C. All cells were authenticated using short tandem repeat DNA fingerprinting and regularly examined for mycoplasma contamination.

The vesicular stomatitis virus (VSV) and Sendai virus (SeV) used in this study were gifted by Professor Y.W. Gao (Changchun Veterinary Research Institute).

### Plasmids

pLVX-Sec8-HA-IRES-Puro, pLVX-Sec8-Flag-IRES-Puro, and pCDNA3.1-HA-STUB1 were obtained from human cDNA libraries using standard PCR techniques and were subsequently inserted into mammalian expression vectors as indicated. The conservative lysines of RIG-I were searched by the UbPred program (http://ubpred.org/). A series of RIG-I mutants (K-to-R) was generated by the Hieff Mut Site-Directed Mutagenesis Kit (Yeasen, 11003ES10) and confirmed by sequencing. All other plasmids, including pLVX-Flag-RIG-I-IRES-Puro, pCDNA3.1-HA-RIG-I, pLVX-Flag-RIG-I-2CARD, pLVX-Flag-RIG-I-Δ2CARD, pLVX-Flag-RIG-I(N)-IRES-Puro, pLVX-Flag-MDA-5(N)-IRES-Puro, pLVX-Flag-MAVS-IRES-Puro, pLVX-Flag-TBK1-IRES-Puro, pLVX-Flag-IRF3-5D-IRES-Puro, pCDNA3.1-Flag-NEDD4, pCDNA3.1-Flag-SMURF2, pCDNA3.1-Myc- Ub, pCDNA3.1-Myc-K6-Ub, pCDNA3.1-Myc-K11-Ub, pCDNA3.1-Myc-K27-Ub, pCDNA3.1-Myc-K29-Ub, pCDNA3.1-Myc-K33-Ub, pCDNA3.1-Myc-K48-Ub, pCDNA3.1-Myc-K63-Ub, pCDNA3.1-Flag-STUB1, pCDNA3.1-Flag-STUB1-TPR, pCDNA3.1-Flag-STUB1-ΔTPR, are stored in He’s lab.

### Generation of shRNA

Sec8 short hairpin RNA (shSec8), STUB1 short hairpin RNA (shSTUB1), and Smurf2 short hairpin RNA (shSmurf2) in Table S1 were designed through the ThermoFisher BLOCK-iT™ RNAi Designer platform (https://rnaidesigner.thermofisher.com), synthesized by Tsingke (Beijing), and ligated into the pYr-Lvsh vector according to the manufacturer’s instructions.

### Antibodies and reagents

Mouse anti-RNF5 (sc-81716) was purchased from Santa Cruz Biotechnology Co., Ltd. Rabbit anti-Sec8 (A12374), Rabbit anti-p53 (A19585), Rabbit anti-CISH (A14527), Rabbit anti-p-IRF3 (S386) (AP0995, used for human), and Mouse anti-FLAG-Tag (AE005) were purchased from Abclonal Biotechnology Co., Ltd. Rabbit anti-β-actin (AB0035), Rabbit anti-DDX58 (RIG-I, used for human) (CY6992), Rabbit anti-IRF3 (CY5779), Rabbit anti-p-IRF3 (S386) (CY6575), Rabbit anti-STUB1 (CY8471), Rabbit anti-p-p53 (Ser33) (CY5083), Rabbit anti-HIF-1 (CY5538), Rabbit anti-AR (CY5030), Rabbit anti-AP-2 (CY1675), Mouse anti-Myc-Tag (AB0001), Rabbit anti-NEDD4 (CY8238), Rabbit anti-Smurf2 (CY8020), and Rabbit anti-RNF125 (AY3939) were obtained from Abways Biotechnology Co., Ltd. Rabbit anti-HA Tag (C29F4) (#3724) and anti-p-IRF3 (S396) (D6O1M, used for mice) were obtained from Cell Signaling Technology. Rabbit anti-K48-linkage specific ubiquitin (YM8266) and Rabbit anti-K63-linkage specific ubiquitin (YM9016) were obtained from Immunoway Biotechnology Co., Ltd. Rabbit anti-DDX58 (RIG-I, used for human or mice) (20566-1-AP) was purchased from Proteintech Biotechnology Co., Ltd. Anti-HA-tag mAb-HRP-DirecT (M180-7), anti-FLAG-tag mAb-HRP-DirecT (M185-7), anti-FLAG-tag Magnetic Beads (M185-11), and anti-HA-tag Magnetic Beads (M180-11) were obtained from MBL Biotechnology Co., Ltd. The secondary antibody AffiniPure Goat Anti-Mouse IgG (H + L) (115-035-003) (AB_10015289) and AffiniPure Goat Anti-Rabbit IgG (H + L) (111-035-003) (AB_2313567) were purchased from Jackson ImmunoResearch Labs. Eosin (C0109), Hematoxylin (C0107), and LPS (S1732) were purchased from the Beyotime Institute of Biotechnology. Poly (I:C) (LMW) (tlrl-picwlv) and Poly (I:C) (HMW) (tlrl-pic) were purchased from InvivoGen. Poly (I:C) (LMW) (tlrl-picwlv) specifically activates RIG-I upon transfection and specifically activates TLR3 upon infection; Poly (I:C) (HMW) (tlrl-pic) specifically activates MDA-5 upon transfection; LPS (S1732) specifically activates TLR4 [45–49]. CHX (HY-12320), PFT-α (HY-15484), MG132 (HY-13259), and CQ (HY-17589A) were purchased from MedChemExpress. DMSO (472301) was obtained from Sigma-Aldrich. M-CSF (RP-8615) was purchased from ThermoFisher. Three-color Pre-stained Protein Marker (WJ103) and Two-color Pre-stained Protein Marker (WJ101) were purchased from Epizyme. Attractene Transfection Reagent (301007) was purchased by QIAGEN. Protein A/G beads (L-1004) were purchased from Biolinkedin Biotechnology Co., Ltd.

### Mice

The Sec8^fl/fl^ (Strain NO. T008897) mice were generated by GemPharmatech (Nanjing, China) using the CRISPR-Cas9 system. The Cre recombinase-mediated deletion of exons 2–9 between the flanking loxp sites results in either an early translational termination of Sec8, leading to a 28-amino acid polypeptide, or destabilization of Sec8 mRNA. Genotyping was performed by PCR with the primers as follows: T008897-F1 5’-CGTTGCTTTCGCCTTGGATCAGTTA-3’, T008897-R1 5’-ATTTAGCTTGTGTGCCTTTGCGTCT-3’. *Lyz2-Cre* mice were gifted from GemPharmatech. Sec8^fl/fl^ mice used in our experiments were Cre-negative mice. The *Lyz2-Cre* Sec8^fl/fl^ mice were generated by mating *Lyz2-Cre* and Sec8^fl/fl^ mice. All mice used in the experiments were randomly grouped and were 6–8 weeks of age. All mice were raised without specific pathogens, as approved by the Scientific Investigation Committee of the College of Life Sciences, Shandong Normal University.

### Preparation and culture of macrophages

To obtain PMs, mice needed to be injected with 6% starch broth once a day for three consecutive days before the experiment, and PMs were obtained by extracting the peritoneal lavage fluid after rinsing the peritoneal cavity with saline 3 days later. PMs were cultured in RPMI 1640 medium (Sparkjade, CF0002) containing 10% FBS and antibiotics (100 mg/mL streptomycin and 100 U/mL penicillin). For the acquisition of BMDMs, bone marrow was blown and washed out of the femur and tibia with cold RPMI 1640, then the cells were sieved using a cell strainer, centrifuged, resuspended, and cultured with RPMI 1640. After induction with granulocyte-macrophage colony-stimulating factor M-CSF (25 ng/mL) for 7 days, the cells were activated and used for subsequent experiments.

### Viral infection in vivo

Six- to eight-week-old mice (male and female) were injected with VSV by tail vein injection for in vivo infection as described previously [50–52]. Mice were weighed 14 days after tail vein injection of VSV (2 × 10^7^ PFU per mouse), and survival rates were determined 7 days after tail vein injection of VSV (1 × 10^8^ PFU per mouse). The mRNA expression of IFN-β, ISG54, ISG56, or VSV-G was detected in the organs of virus-infected mice via qPCR. At the specified time, H&E staining of the lung was performed on virus-infected mice. The protein level of IFN-β in the serum of mice infected with VSV was determined by ELISA Kit (MultiSciences Biotech, EK2236-96).

### Viral infection in vitro

Cells were infected with VSV or SeV in serum-free culture sets for 1 h, followed by rinsing twice with PBS (Sparkjade, CR0014-500Ml) and replacing with fresh medium (2% FBS) according to the procedure described previously [53]. Cells or supernatants were analyzed by TCID_50_, qPCR, and western blotting after collection at the specified time points.

### Construction of RIG-I or STUB1 knockout stable cell line

The construction of knockout cell lines was performed as previously described [54]. Briefly, RIG-I or STUB1 knockout stable cell lines were constructed using the CRISPR/Cas9 system. Targeting sgRNAs for human RIG-I or STUB1 was designed using the mized Crispr Design program (http://crispr.mit.edu/), with sequences listed in Table S1. Lentivirus (Lenti-CAS9-sgRNA-puro) encoding Cas9 and the sgRNAs for RIG-I or wild-type (WT) control was produced by Tsingke (Beijing). HeLa cells were infected with the lentivirus and selected with 2.0 μg/mL puromycin (Yeasen, 60210ES25) after 72 h to obtain stable knockout cell lines.

### RNA extraction and qPCR

RNA extraction and reverse transcription were performed as previously [55]. Quantitative PCR (qPCR) was used to measure the levels of mRNA, as explained by Hou et al. [56]. The 2^−ΔΔCT^ method was used to calculate the relative fold change in mRNA expression [57]. All primers were designed by Primer Premier 6.0 software, with primer sequences shown in Table S1. Total RNA Extraction Kit (Sparkjade, AC0205-B), RT Mix Kit with gDNA Clean for qPCR Ver.2 (Accurate Biotech, AG11728) and 2X SYBR Green Pro Taq HS Premix (Accurate Biotech, AG11701) were used in this paper.

### Chromatin immunoprecipitation (CHIP)

CHIP assays were performed as described previously [58] and conducted in the ChIP Assay Kit (Beyotime Biotech, P2078). Briefly, cells are fixed with formaldehyde (Chronchem, 50-00-0), followed by lysis and chromatin shearing to create 200–600 bp fragments. The solution is incubated with the p53 antibody and then with magnetic beads for immunoprecipitation. Following extensive washing to remove nonspecific interactions, cross-linking is reversed to purify the bound DNA. The enriched DNA is analyzed by PCR using specific primers (F: 5’-GCCCTCAGCGAAGCAAGTG-3’, R: 5’-TGGGAGGAACGCTCCAGTT-3’) to quantify target sequences, indicating the interaction between the protein and DNA.

### TCID_50_

First, suitable cell lines (e.g., HeLa) were cultured in 96-well plates until 80% confluent. Then virus stocks were tenfold serially diluted and added to the wells in octuplicate. After incubation for 72 h at 37 °C with 5% CO_2_, the presence of cytopathic effects was observed. The TCID_50_ was calculated using the Reed-Muench method or the Spearman-Karber method.

### Immunoprecipitation

Immunoprecipitation assays were performed as described previously [59]. For immunoprecipitation, the cells cultured in 100 mm cell culture dishes (BaiDi Biotech, H802007) were harvested with cell scrapers (Jet Biofil, CSC211023). Then the cells were lysed with Native lysis buffer (Solarbio, R0030) supplemented with protease and phosphatase inhibitor cocktail (Thermo Fisher, 87786) for 30 min. After centrifugation, cell lysates were incubated with the indicated antibody-conjugated beads. Subsequently, the beads were washed ~5–6 times and then subjected to Western blotting analysis.

### Western blot

Western blot assays were performed as described previously [59]. Briefly, the cells were harvested and lysed in RIPA buffer (NCM Biotech, WB3100) supplemented with protease and phosphatase inhibitor cocktails for 30 min on ice. After the supernatant was denatured by adding SDS-PAGE protein sampling buffer (Beyotime, P0015), protein samples were separated by SDS polyacrylamide gel electrophoresis (NCM Biotech, P2011, P2012, or P2013) and transferred to PVDF membrane (Millipore, IPVH00010). The membranes were blocked with 5% skimmed milk and then incubated with the corresponding primary antibody and the secondary antibody. Finally, the immunoreactive signals were detected by using the Omni-ECL™ Femto Light Chemiluminescence Kit (Sparkjade, ED0015-C).

### Dual-luciferase reporter assay

Dual-luciferase reporter assays were performed as described previously [60]. HEK-293T cells were seeded into 24-well plates (Jet Biofil, Guangzhou, China, TCP011024) and co-transfected with pRL-TK plasmid (a Renilla luciferase plasmid; 10 ng), firefly luciferase (100 ng), and appropriate plasmids for 24 h. Then, cells were harvested, and the firefly and Renilla luciferase activities were measured using the Dual-Luciferase Reporter Analysis System Kit (Vazyme Biotech, DD1205-01) with the SpectraMax M5 microplate reader (Molecular Devices Instruments Inc., USA). The measured fluorescence intensities of firefly luciferase were normalized with Renilla luciferase to determine the relative luciferase activity.

#### Quantification and statistical analysis

GraphPad Prism (version 8.0, GraphPad, San Diego, CA, USA) software was used for statistical analyses. Unpaired two-sided Student’s *t* test, ordinary one-way ANOVA, and two-way ANOVA were utilized to analyze the significance of between-group differences, as well as the log-rank (Mantel–Cox) test for mouse survival curve analysis. Analyze the gray-scale intensity of the western blot bands displayed using ImageJ software. For mouse survival data, the Kaplan‒Meier curve was employed. Data are expressed as mean value ± SD (standard deviation) of at least three independent experiments. Significance levels were set as follows: Ns: no significant difference, *p* > 0.05; *, *p* < 0.05; **, *p* < 0.01; ***, *p* < 0.001.

## Supplementary information

Supplementary index

Original source data for Western blot

## Data Availability

The authors declare that all data on which the conclusions of this study are based can be found in the paper and its Supplementary Information document, or can be obtained from the corresponding authors upon reasonable request.
