# Supplementary material for: Sec8: a novel positive regulator of RIG-I in anti-RNA viral defense
Source: Cell Death Dis. 2026 Jan 24;17(1):165. doi: 10.1038/s41419-026-08414-9 (PMC12877145; doi:10.1038/s41419-026-08414-9)
Supplement: Supplementary file 1 — Supplementary index [file 41419_2026_8414_MOESM1_ESM.docx]

**Supplemental information for CDDIS-25-1337R
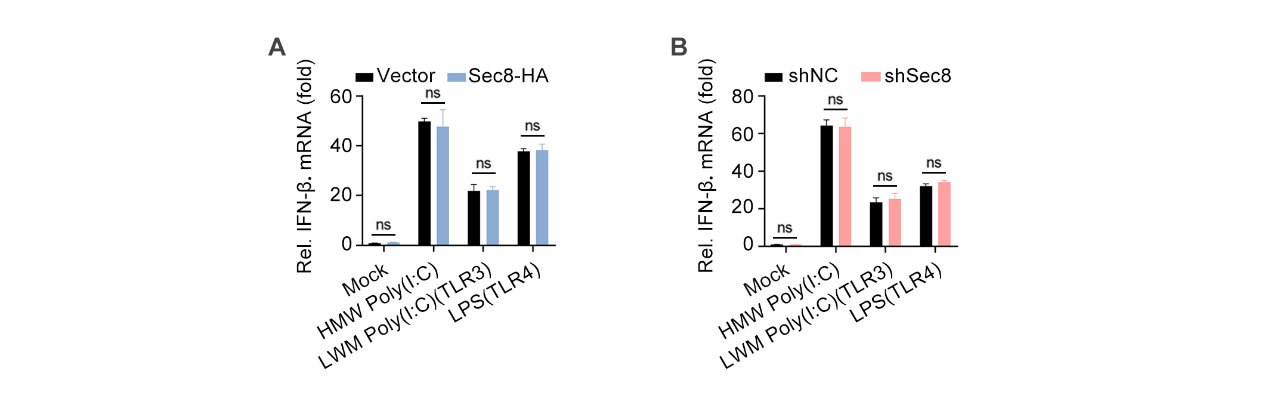
**

**Supplementary** **Figure 1** **Analysis of the effects of Sec8 on different natural immune signaling pathways.**

**(A)** qPCR analysis of the mRNA expression of IFN-β in Sec8-overexpressing HeLa cells transfected with HMW poly(I:C) (10 μg/mL), infected with the agonists of TLR3 (poly I:C) (10 μg/mL) or TLR4 (LPS) (5 μg/mL) for 12 h. **(B)** qPCR analysis of the mRNA expression of IFN-β in Sec8-silencing HeLa cells transfected with HMW poly(I:C) (10 μg/mL), infected with the agonists of TLR3 (poly I:C) (10 μg/mL) or TLR4 (LPS) (5 μg/mL) for 12 h. Significance differences were determined by two-way ANOVA, with significance levels denoted as follows: ns, not significant.

**
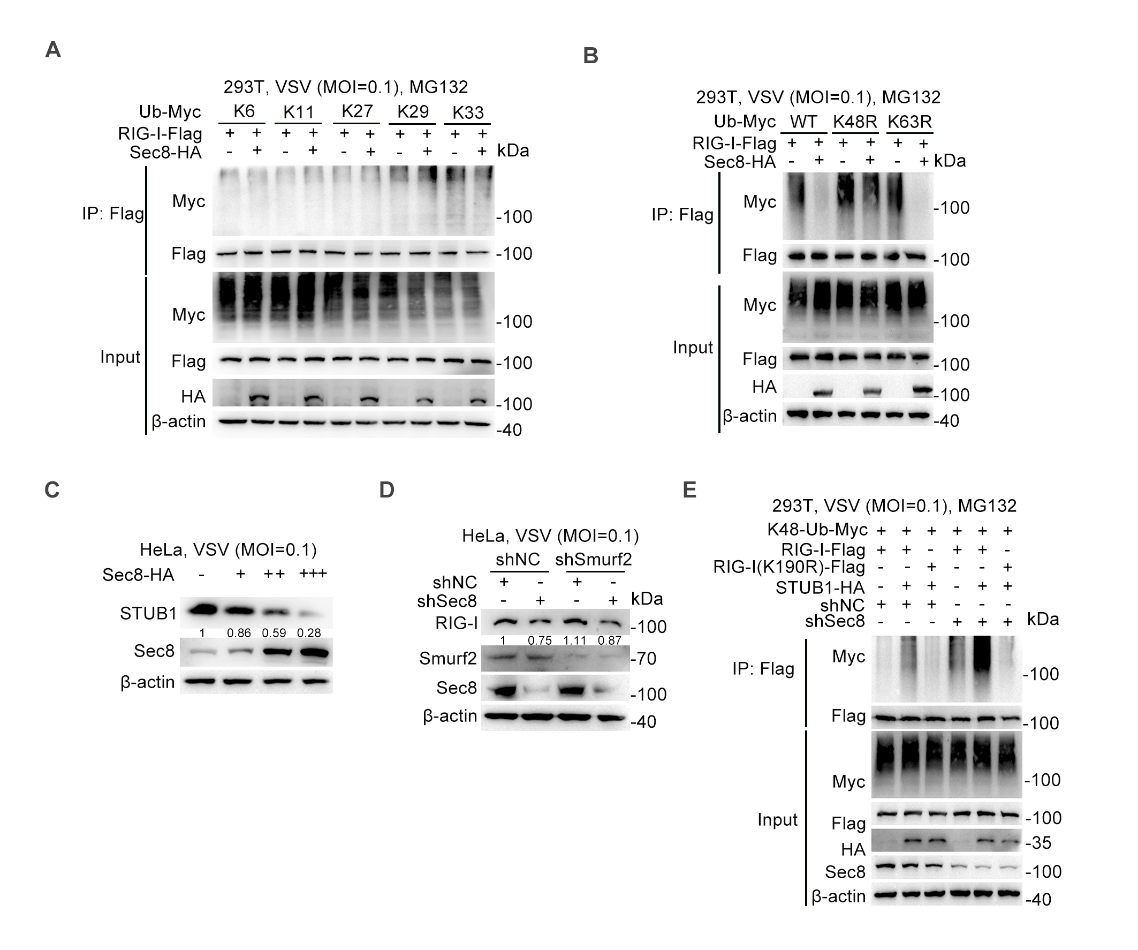
**

**Supplementary Figure 2 Sec8 inhibits STUB1-mediated ubiquitin-proteasome degradation of RIG-I at K190.**

**(A)** Evaluation of various lysine ubiquitination types (K6, K11, K27, K29, and K33) on RIG-I by Sec8 was performed by co-transfecting HEK-293T cells with relevant plasmids, followed by MG132 treatment and VSV infection (MOI=0.1) for 12 hours. **(B)** Evaluation of various lysine ubiquitination types (wild type, K48R, and K63R) on RIG-I was performed by co-transfecting HEK-293T cells with relevant plasmids, followed by MG132 treatment and VSV infection (MOI=0.1) for 12 hours. **(C)** Immunoblot analysis of STUB1 expression following transfection with varying concentrations of Sec8-HA (0.6, 1.2, or 1.8 μg/mL) or empty plasmid in HeLa cells infected with VSV. **(D)** Immunoblot analysis of RIG-I protein level in Sec8-silencing HeLa cells transfected with shSmurf2 or negative control (shNC). **(E)** Immunoprecipitation analysis of the K48-linkage polyubiquitin ubiquitination of RIG-I from Sec8-silencing cells transiently cotransfected with STUB1, along with RIG-I (wild-type or K190R) in HEK-293T cells, followed by MG132 treatment and VSV infection.

**
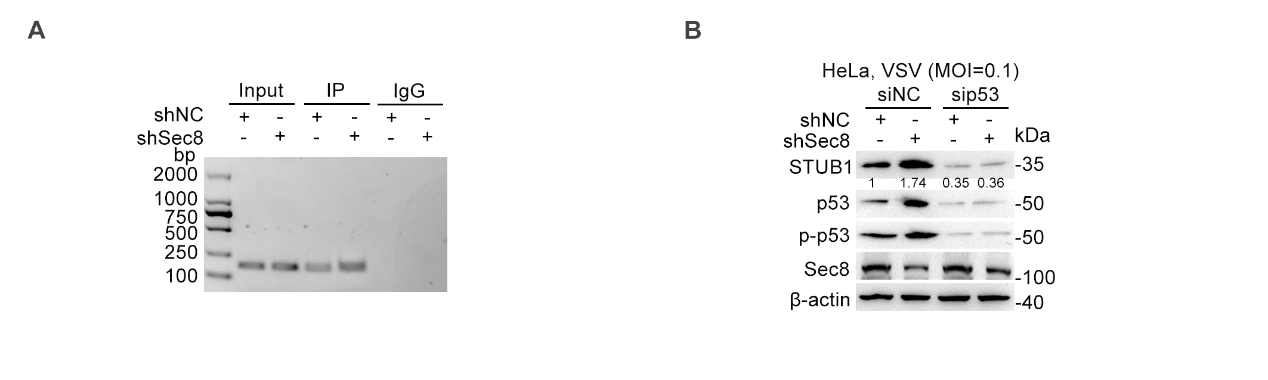
****Supplementary Figure 3 Sec8 inhibits the p53-mediated transcription of STUB1.**

**(A)** ChIP was performed in HEK-293T cells with p53 antibody. The effect of Sec8 silencing on the interaction between the p53 protein and the STUB1 promoter region was analyzed by PCR with STUB1-designated primers. **(B)** Immunoblot analysis of STUB1 in control or Sec8-silencing HeLa cells treated with control siRNA or p53 siRNA and infected with VSV (MOI=0.1) for 12 hours.


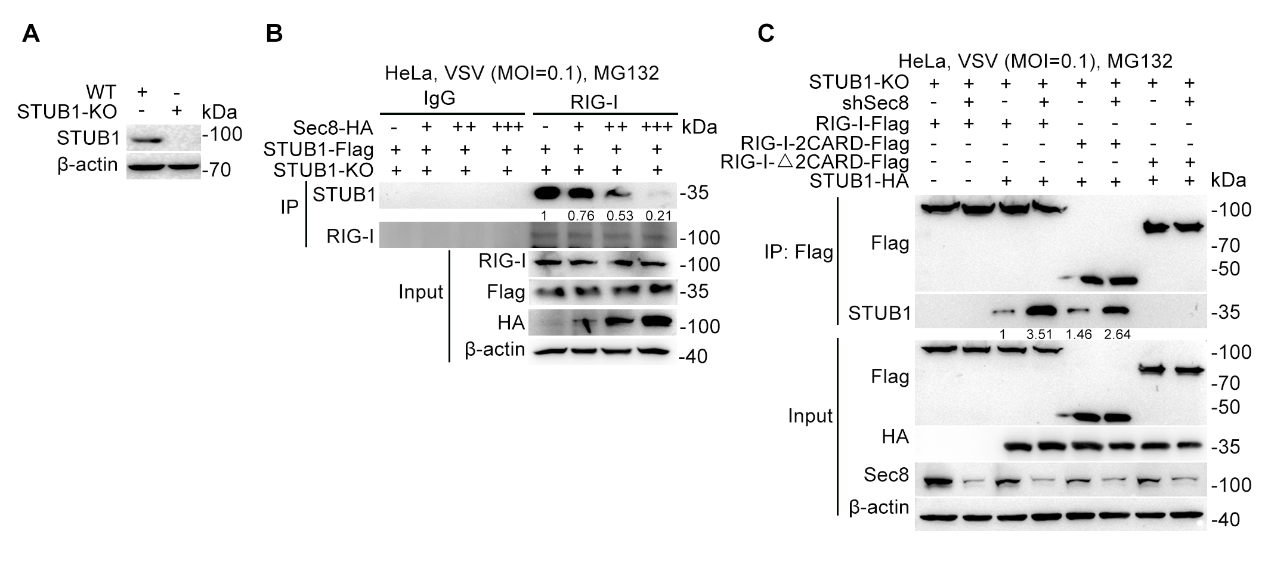


**Supplementary Figure 4 Sec8 competitively binds the 2CARD domain of RIG-I with STUB1.**

**(A)** Immunoblot analysis of STUB1 knockout efficiency in STUB1-KO HeLa cell lines. **(B)** Transfect STUB1-Flag into STUB1-KO HeLa cell lines, simultaneously co-transfecting with different concentrations of Sec8-HA (0.6, 1.2, or 1.8 μg/mL) or empty plasmid, followed by MG132 treatment and VSV infection (MOI=0.1) for 12 hours. Subsequently, immunoprecipitation analysis of the interaction between STUB1 and RIG-I was performed. **(C)** Transfected STUB1-Flag into STUB1-KO HeLa cell lines, simultaneously co-transfected with either full-length RIG-I or its domain mutants (RIG-I-2CARD and RIG-I-△2CARD) alongside shSec8 or empty vector, followed by treatment with MG132 and infected with VSV (MOI=0.1) for 12 hours. Subsequently, immunoprecipitation analysis was performed to detect interactions between STUB1 and RIG-I or its domain mutants.

**Table S1. sequences used in this study**

| **Primer Name** | **Primer Sequences (5’ to 3’)** | **Application** |
| --- | --- | --- |
| VSV-G-F | GATGAATACACAGGAGAATGG | qPCR for VSV-G |
| VSV-G-R | ATGGAGCAGAGATACTTGAC |  |
| SeV-NP-F | TGCCCTGGAAGATGAGTTAG | qPCR for SeV-NP |
| SeV-NP-R | GCCTGTTGGTTTGTGGTAAG |  |
| Human-IFN-β-F | CAGCAATTTTCAGTGTCAGAAGCT | qPCR for Human-IFN-β |
| Human-IFN-β-R | TCATCCTGTCCTTGAGGCAGTAT |  |
| Human-ISG54-F | AGGACACGCTGTGGCTCATCT | qPCR for Human-ISG54 |
| Human-ISG54-R | GGAGGCTGGCAAGAATGGAACA |  |
| Human-ISG56-F | TGGACAAGGTGGAGAACA | qPCR for Human-ISG56 |
| Human-ISG56-R | CAGGCGATAGGCAGAGAT |  |
| Human-β-actin-F | CGAGAAGATGACCCAGAT | qPCR for Human-β-actin |
| Human-β-actin-R | GATAGCACAGCCTGGATA |  |
| Mouse-IFN-β-F | CAGCTCCAAGAAAGGACGAAC | qPCR for Mouse-IFN-β |
| Mouse-IFN-β-R | GGCAGTGTAACTCTTCTGCAT |  |
| Mouse-ISG54-F | GCCATTGCGAACTACCGTCTGG | qPCR for Mouse-ISG54 |
| Mouse-ISG54-R | GGCTGCCCTGAGGAGTGTATCT |  |
| Mouse-ISG56-F | GGCTGGAGTGTGCTGAGATGGA | qPCR for Mouse-ISG56 |
| Mouse-ISG56-R | CTTGGCGATAGGCTACGACTGC |  |
| Mouse-β-actin-F | CCACACCCGCCACCAGTTCG | qPCR for Mouse-β-actin |
| Mouse-β-actin-R | TACAGCCCGGGGAGCATCGT |  |
| Human-RIG-I-F | ACCAGAGGCAGAGGAAGAGCAA | qPCR for Human-RIG-I |
| Human-RIG-I-R | TCGTCCCATGTCTGAAGGCGTA |  |
| Human-STUB1-F | TCCTACCTCTCCAGGCTCATTGC | qPCR for Human-STUB1 |
| Human-STUB1-R | ATGTCCGCCATGTACTTGTCGTG |  |
| Human-RIG-I-sgRNA-F | CACCGCAGGCTGCGTCGCTGCTCGG | LentiCRISPR-V2-RIG-I |
| Human-RIG-I-sgRNA-R | AAACCCGAGCAGCGACGCAGCCTGC |  |
| Human-STUB1-sgRNA-F | CACCGTCTTGCCACACAGGTAGTCG | LentiCRISPR-V2-STUB1 |
| Human-STUB1-sgRNA-R | AAACCGACTACCTGTGTGGCAAGAC |  |
| Human-STUB1-shRNA-F | GATCCGGCCTTGTGCTACCTGAAGATCTCGAGATCTTCAGGTAGCACAAGGCCTTTTTG | Knockdown of STUB1 |
| Human-STUB1-shRNA-R | AATTCAAAAAGGCCTTGTGCTACCTGAAGATCTCGAGATCTTCAGGTAGCACAAGGCCG |  |
| Human-Sec8-shRNA-F | GATCCGGTCCTGATGACAACTTAATTCTCGAGAATTAAGTTGTCATCAGGACCTTTTTG | Knockdown of Sec8 |
| Human-Sec8-shRNA-R | AATTCAAAAAGGTCCTGATGACAACTTAATTCTCGAGAATTAAGTTGTCATCAGGACCG |  |
| Human-Smurf2-shRNA-F | GATCCCAGTTAATCCGGAACATTTCTCGAGAAATGTTCCGGATTAACTGTTTTTG | Knockdown of Smurf2 |
| Human-Smurf2-shRNA-R | AATTCAAAAACAGTTAATCCGGAACATTTCTCGAGAAATGTTCCGGATTAACTGG |  |
| Human-p53-siRNA-F | UGGUUCACUGAAGACCCAGUU | Knockdown of p53 |
| Human-p53-siRNA-F | UUACCAAGUGACUUCUGGGUC |  |
| Sec8-HA-F | CCGGAATTCGCCACCATGGCGGCAGAAGCAGCTGGT | pLVX-Sec8-HA-IRES-Puro |
| Sec8-HA-R | CCGCTCGAGCTAAGCGTAGTCTGGGACGTCGTATGGGTAAACGGTAGTTATCTTCTT |  |
| Sec8-Flag-F | CCGGAATTCGCCACCATGGCGGCAGAAGCAGCTGGT | pLVX-Sec8-Flag-IRES-Puro |
| Sec8-Flag-R | CCGCTCGAGCTACTTATCGTCGTCATCCTTGTAATCAACGGTAGTTATCTTCTT |  |
| STUB1-HA-F | CCGGAATTCGCCACCATGAAGGGCAAGGAGGAGAAG | pCDNA3.1-HA-STUB1 |
| STUB1-HA-R | CCGCTCGAGTCAAGCGTAGTCTGGGACGTCGTATGGGTAGTAGTCCTCCACCCAGCC |  |
| STUB1-F | GCCCTCAGCGAAGCAAGTG | CHIP for STUB1 |
| STUB1-R | TGGGAGGAACGCTCCAGTT |  |
