# Supplementary material for: Sec8: a novel positive regulator of RIG-I in anti-RNA viral defense
Source: Cell Death Dis. 2026 Jan 24;17(1):165. doi: 10.1038/s41419-026-08414-9 (PMC12877145; doi:10.1038/s41419-026-08414-9)
Supplement: Supplementary file 2 — Original source data for Western blot [file 41419_2026_8414_MOESM2_ESM.pdf]

Source data for:
Sec8: A Novel Positive Regulator of RIG-I in Anti-RNA Viral Defense

Fig. 2C

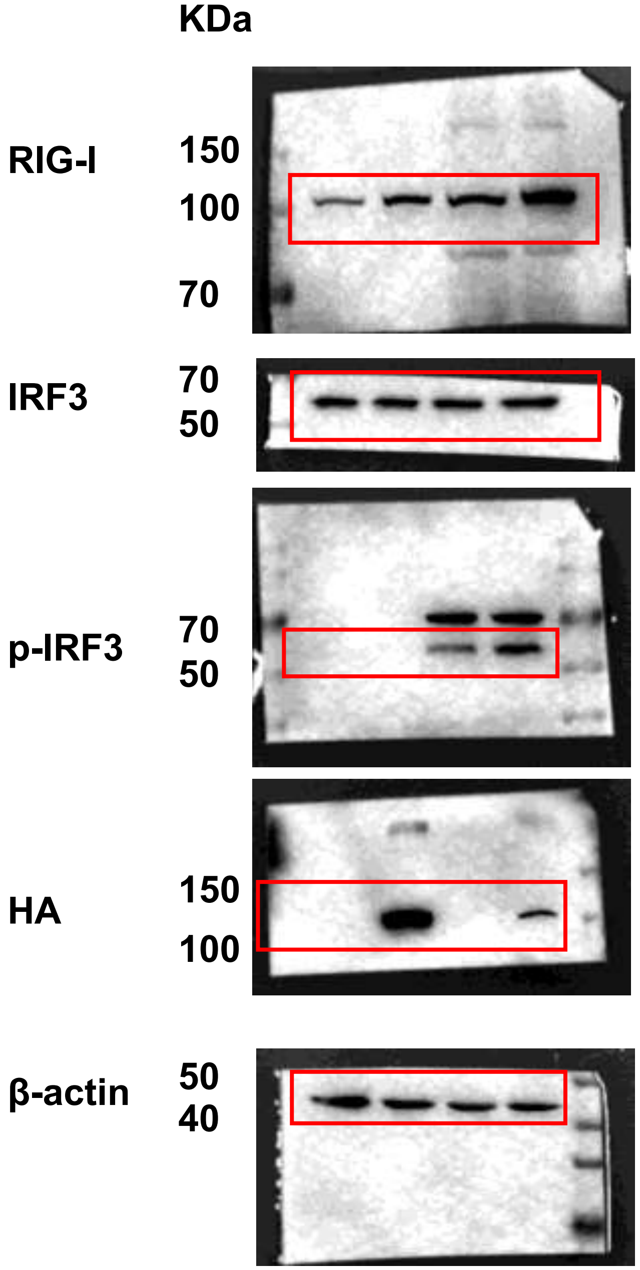
KDa
RIG-I
150
100
70
IRF3
70
50
p-IRF3
70
50
HA
150
100
β-actin
50
40

Fig. 2D

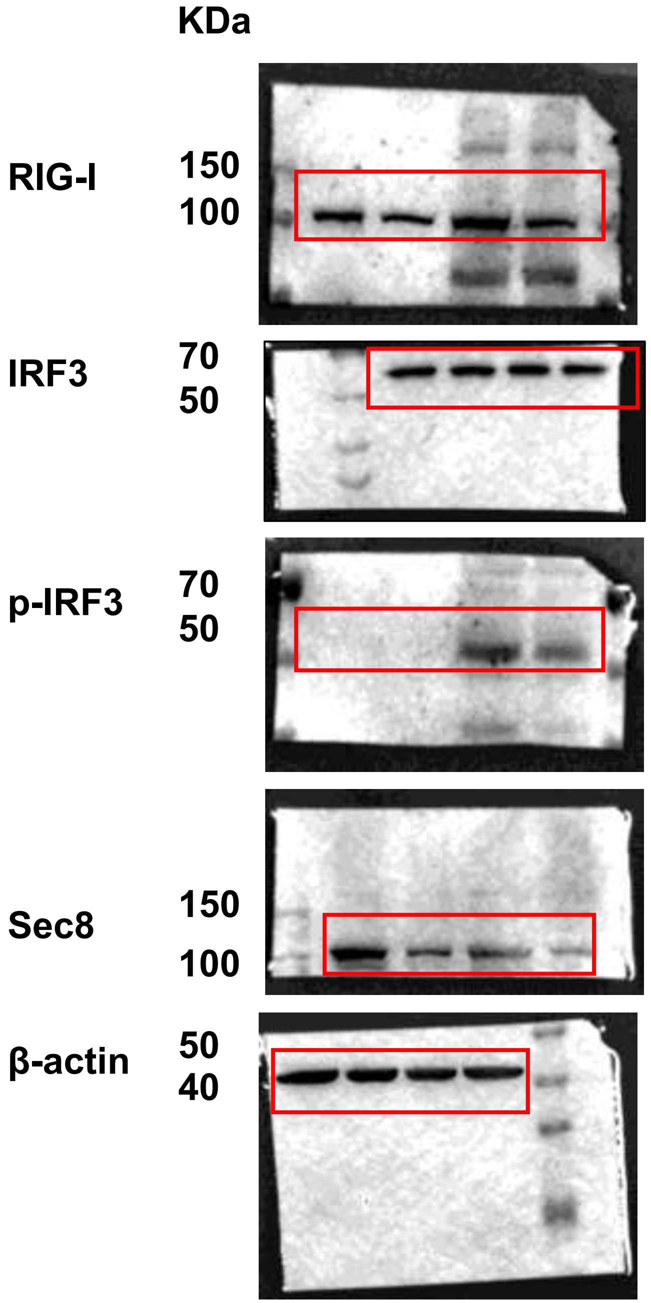
KDa
RIG-I
150
100
IRF3
70
50
p-IRF3
70
50
Sec8
150
100
β-actin
50
40

Fig. 2E

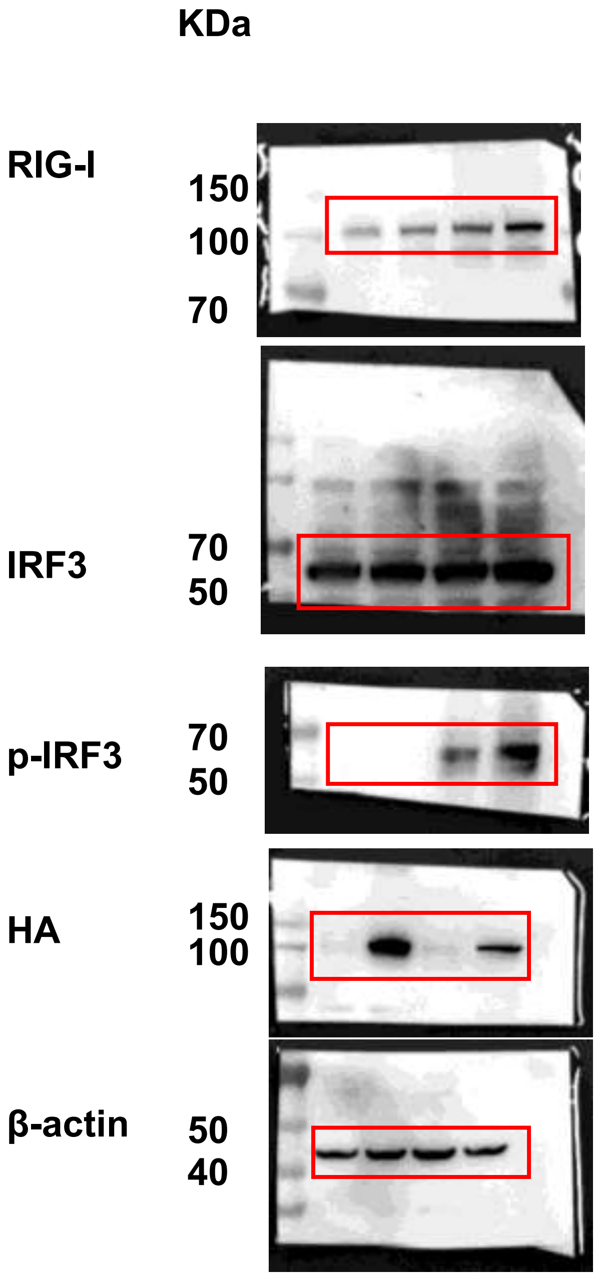

KDa
RIG-I
150
100
70
IRF3
70
50
p-IRF3
70
50
HA
150
100
β-actin
50
40

Fig. 2F

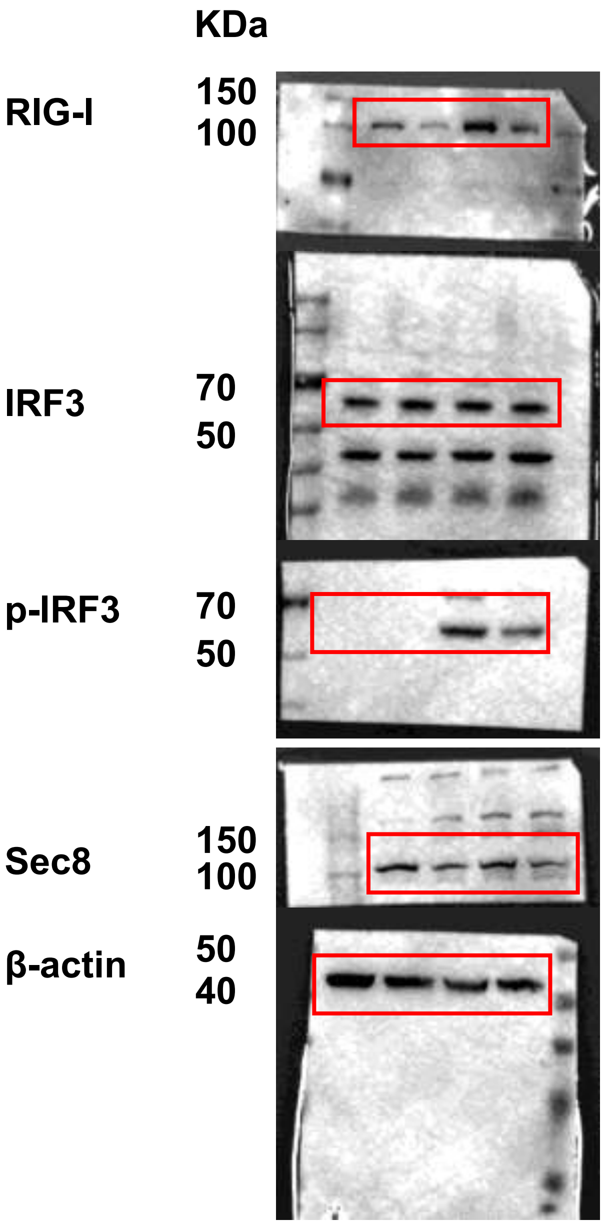

KDa
RIG-I
150
100
IRF3
70
50
p-IRF3
70
50
Sec8
150
100
β-actin
50
40

Fig. 2G

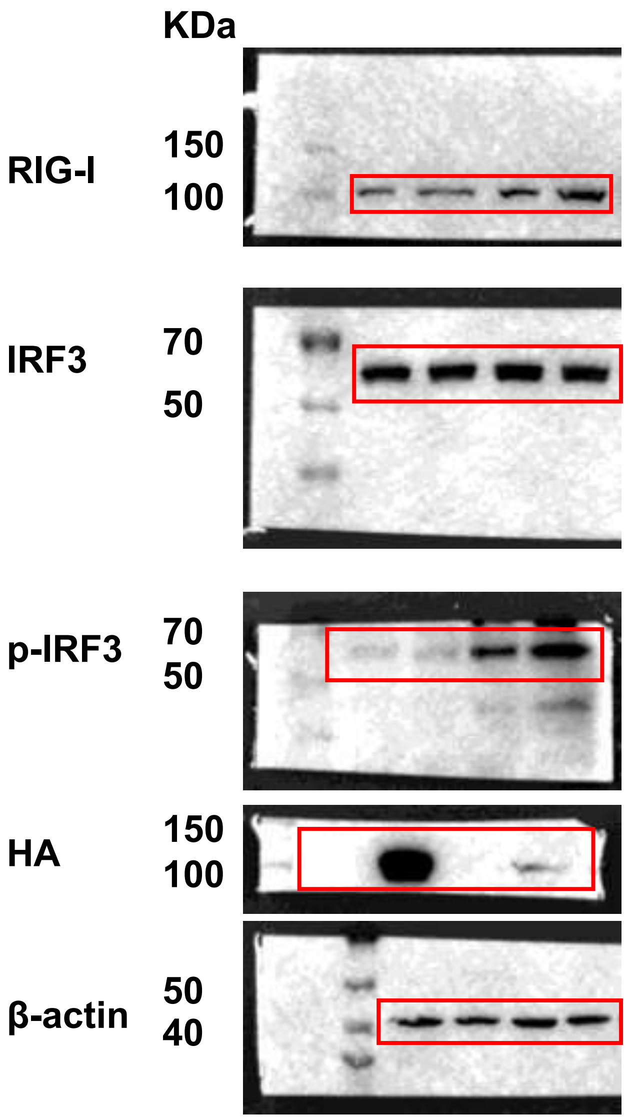

KDa
RIG-I
150
100
IRF3
70
50
p-IRF3
70
50
HA
150
100
β-actin
50
40

Fig. 2H

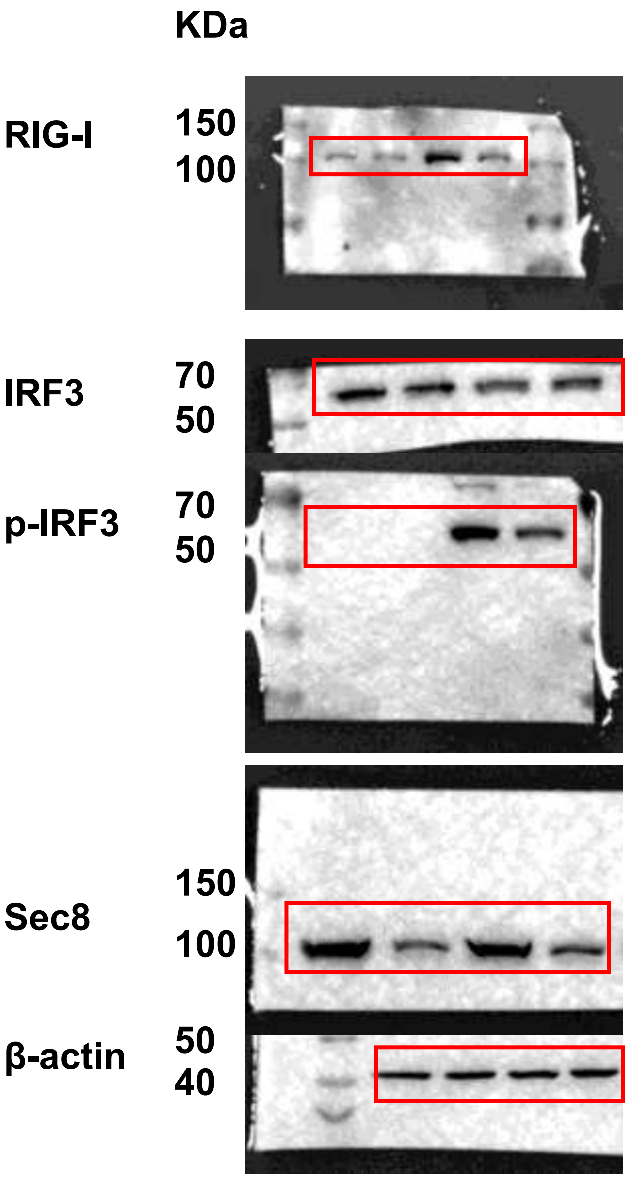

KDa
RIG-I
150
100
IRF3
70
50
p-IRF3
70
50
Sec8
150
100
β-actin
50
40

Fig. 2I

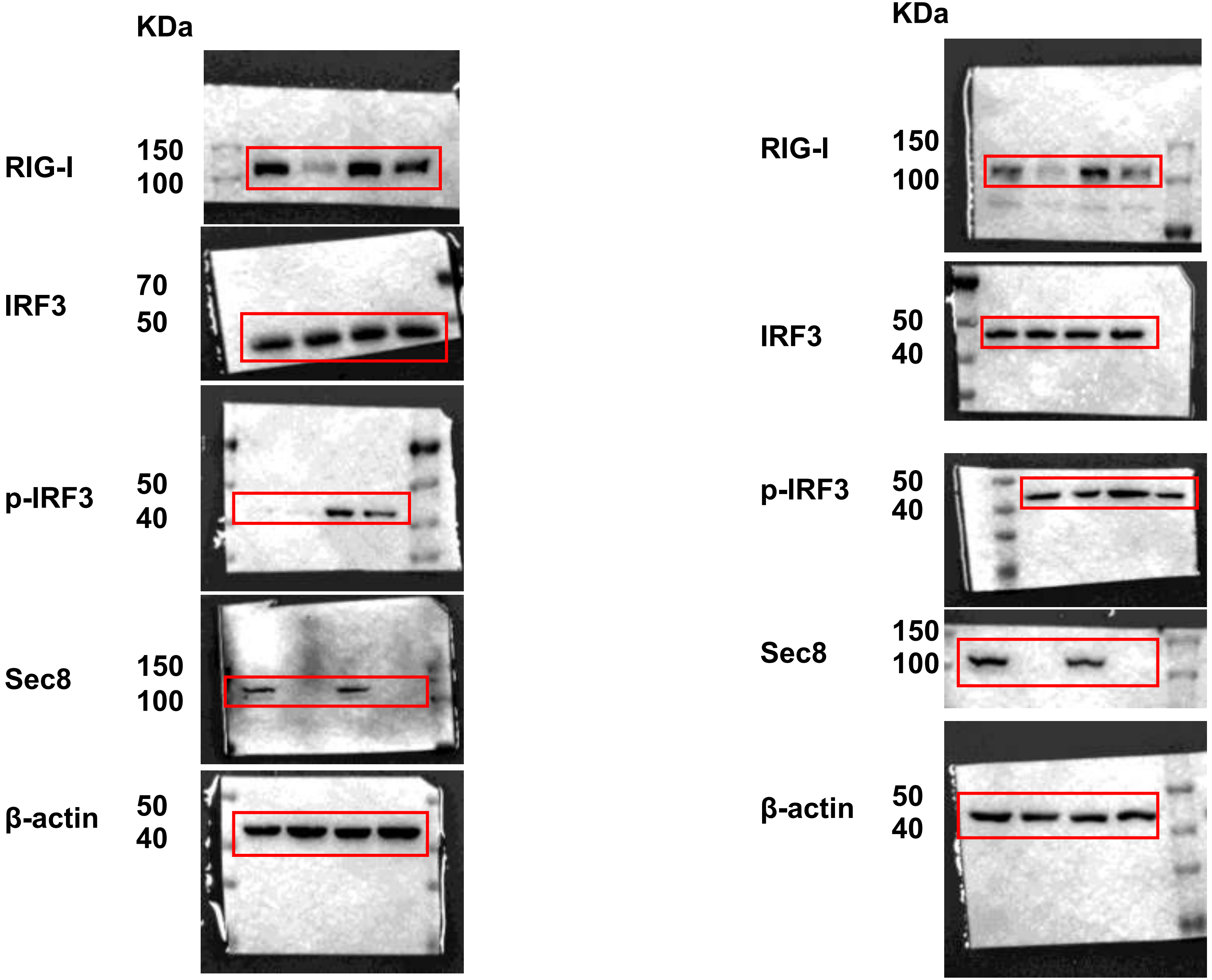
KDa
RIG-I
150
100
IRF3
70
50
p-IRF3
50
40
Sec8
150
100
β-actin
50
40
KDa
RIG-I
150
100
IRF3
50
40
p-IRF3
50
40
Sec8
150
100
β-actin
50
40

Fig. 2J

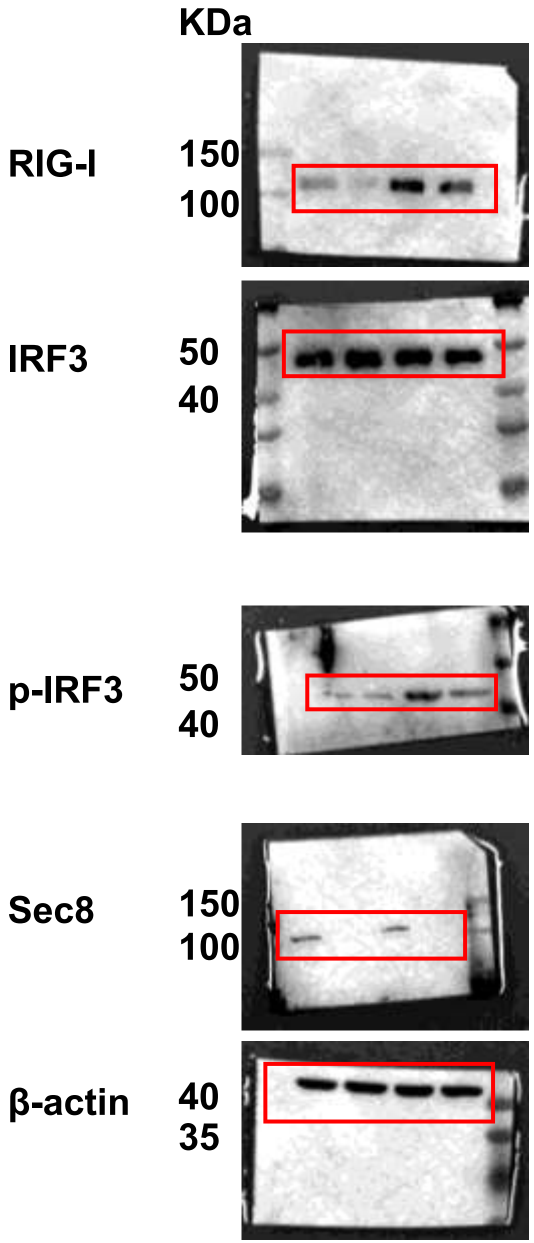
KDa
RIG-I
150
100
IRF3
50
40
p-IRF3
50
40
Sec8
150
100
β-actin
40
35

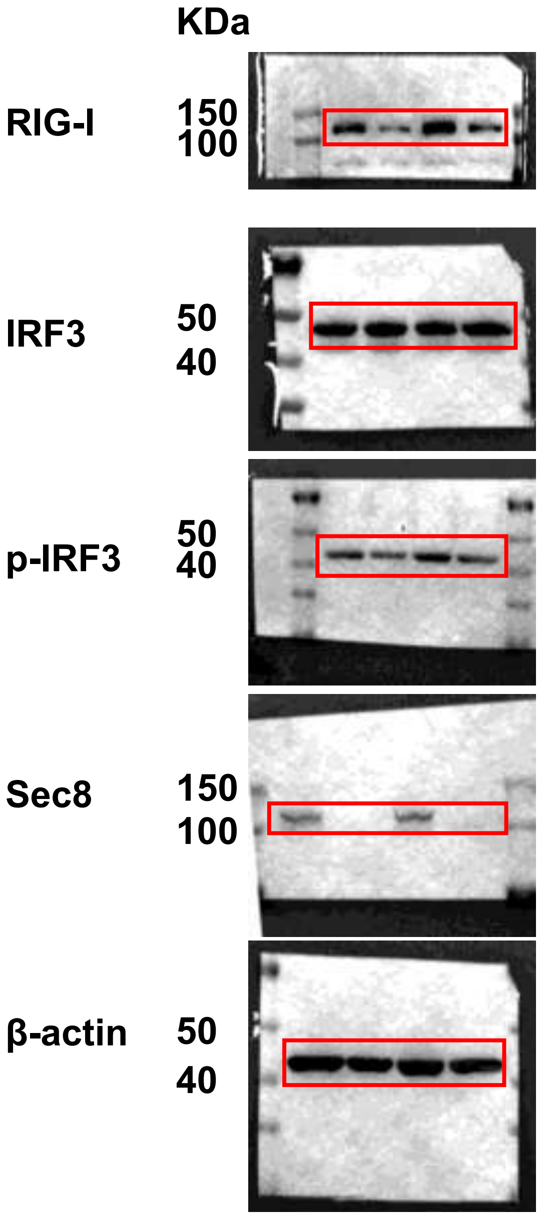
KDa
RIG-I
150
100
IRF3
50
40
p-IRF3
50
40
Sec8
150
100
β-actin
50
40

Fig. 3B

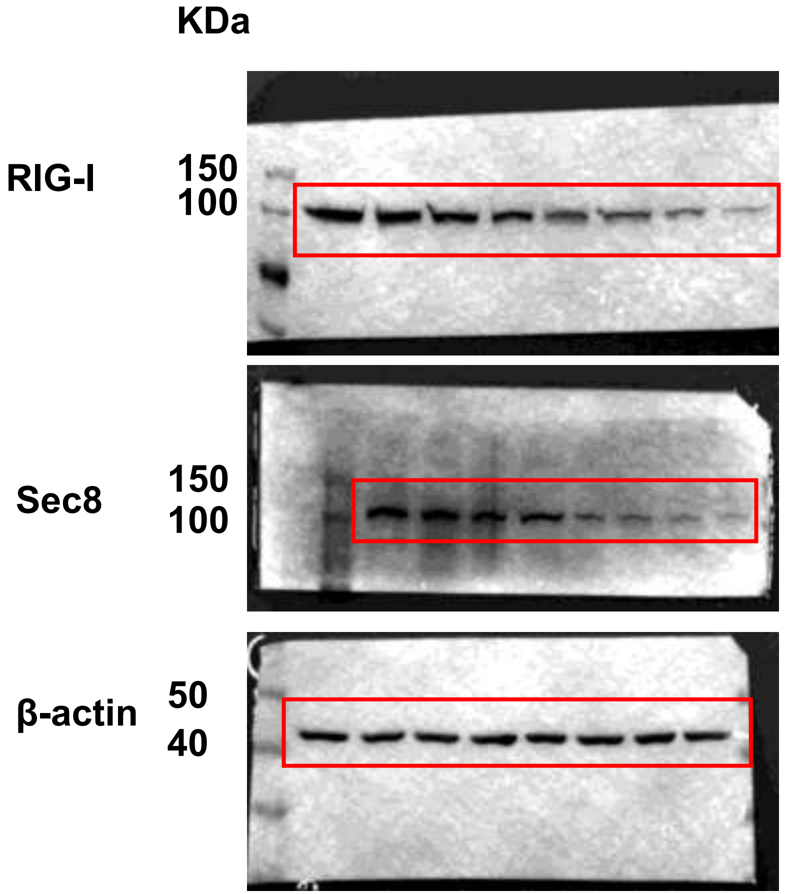
KDa
RIG-I
150
100
Sec8
150
100
β-actin
50
40

Fig. 3D

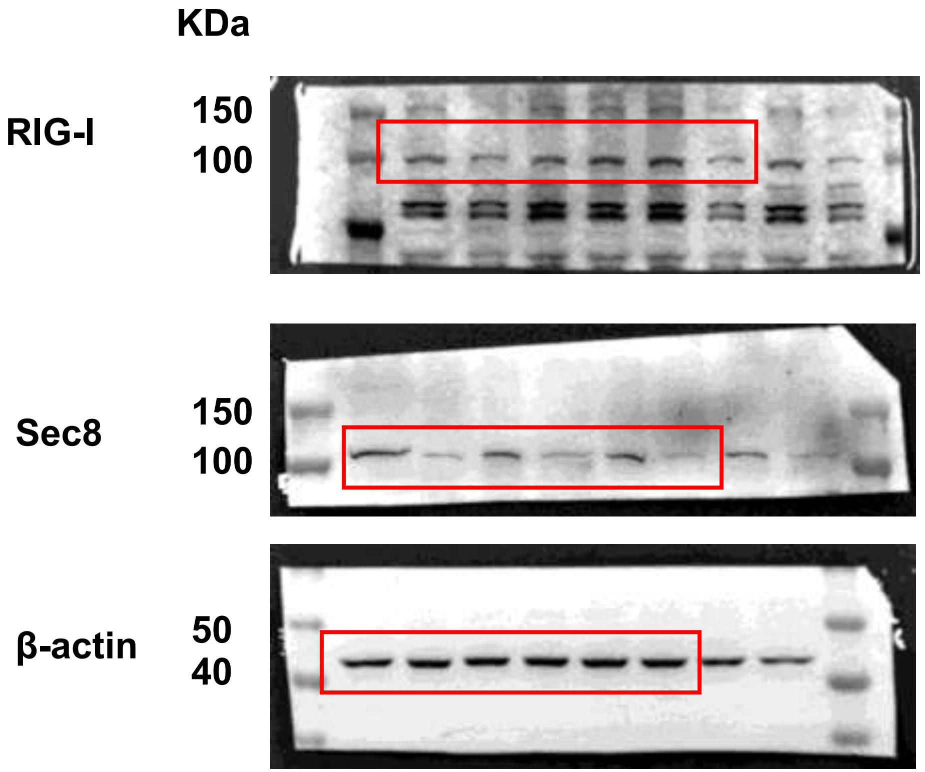
KDa
RIG-I
150
100
Sec8
150
100
β-actin
50
40

Fig. 3E

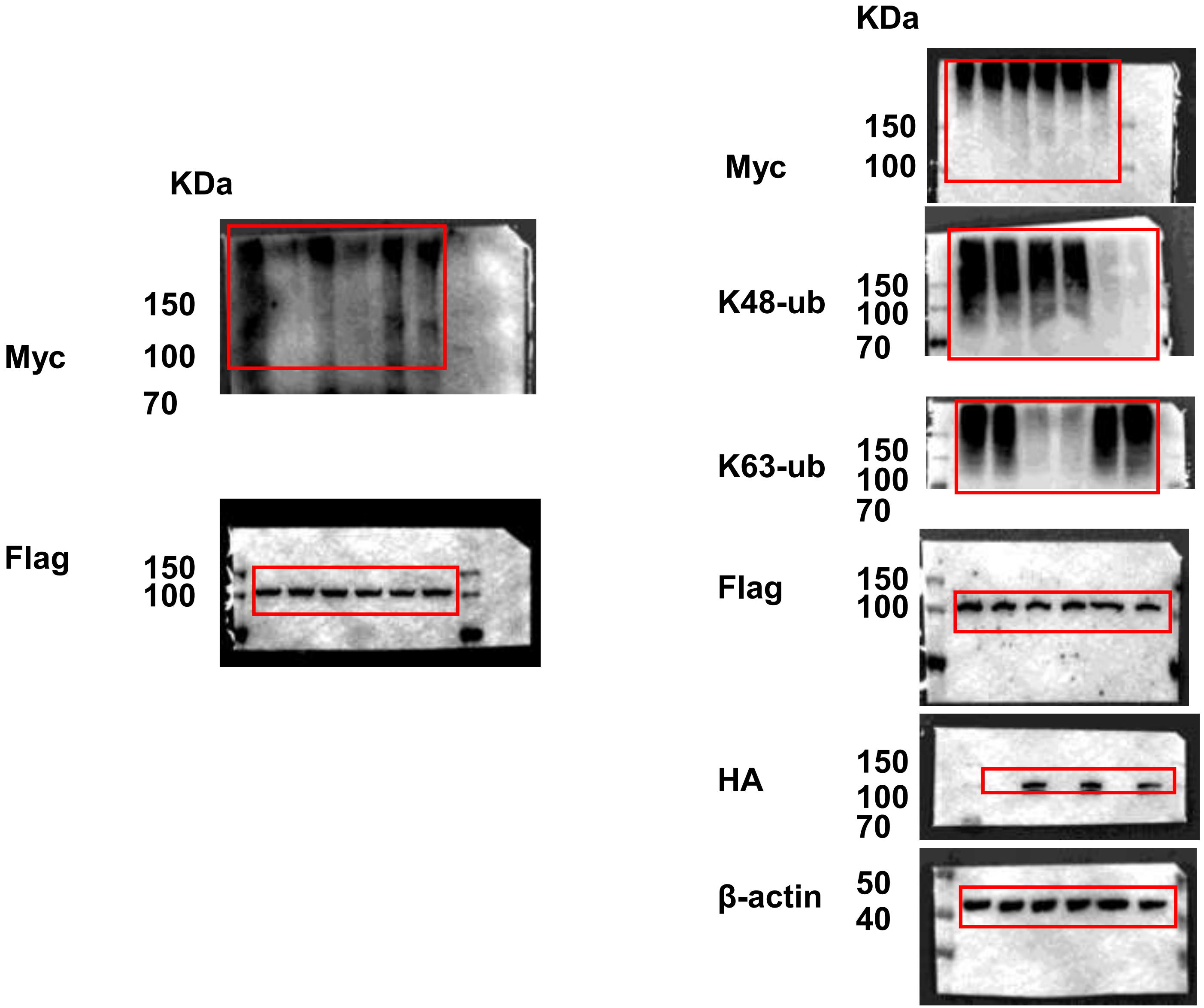

KDa
Myc
150
100
70
Flag
150
100
KDa
Myc
150
100
K48-ub
150
100
70
K63-ub
150
100
70
Flag
150
100
HA
150
100
70
β-actin
50
40

Fig. 3F

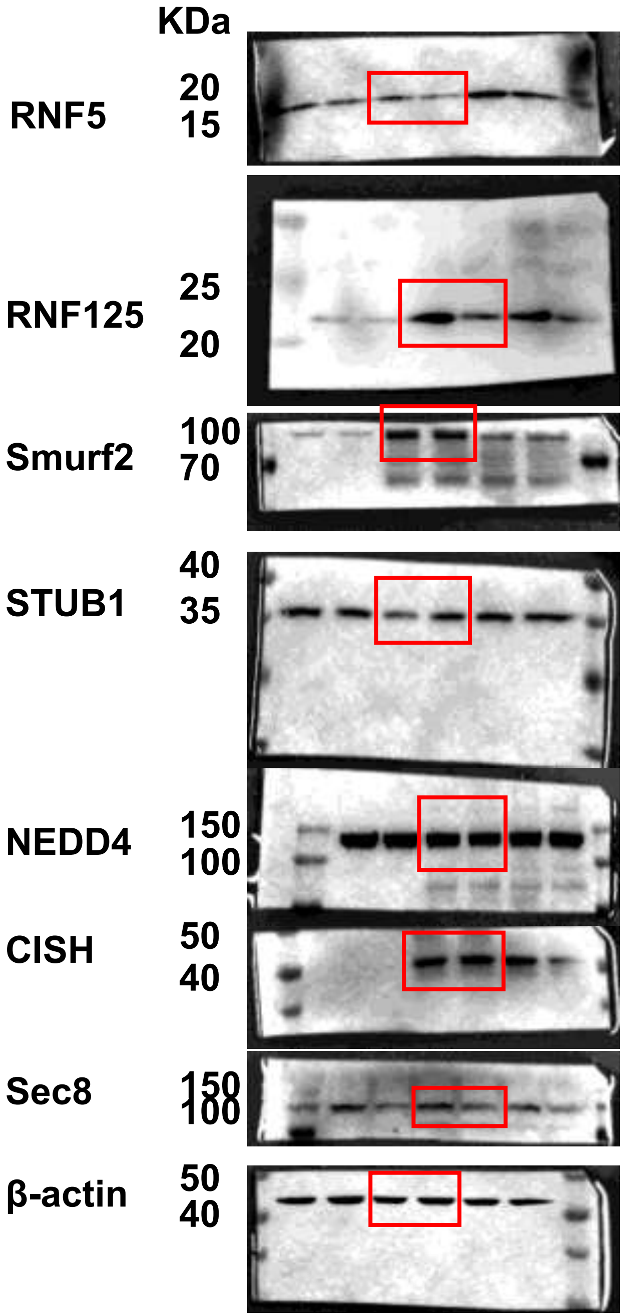

KDa
RNF5
20
15
RNF125
25
20
Smurf2
100
70
STUB1
40
35
NEDD4
150
100
CISH
50
40
Sec8
150
100
β-actin
50
40

Fig. 3G

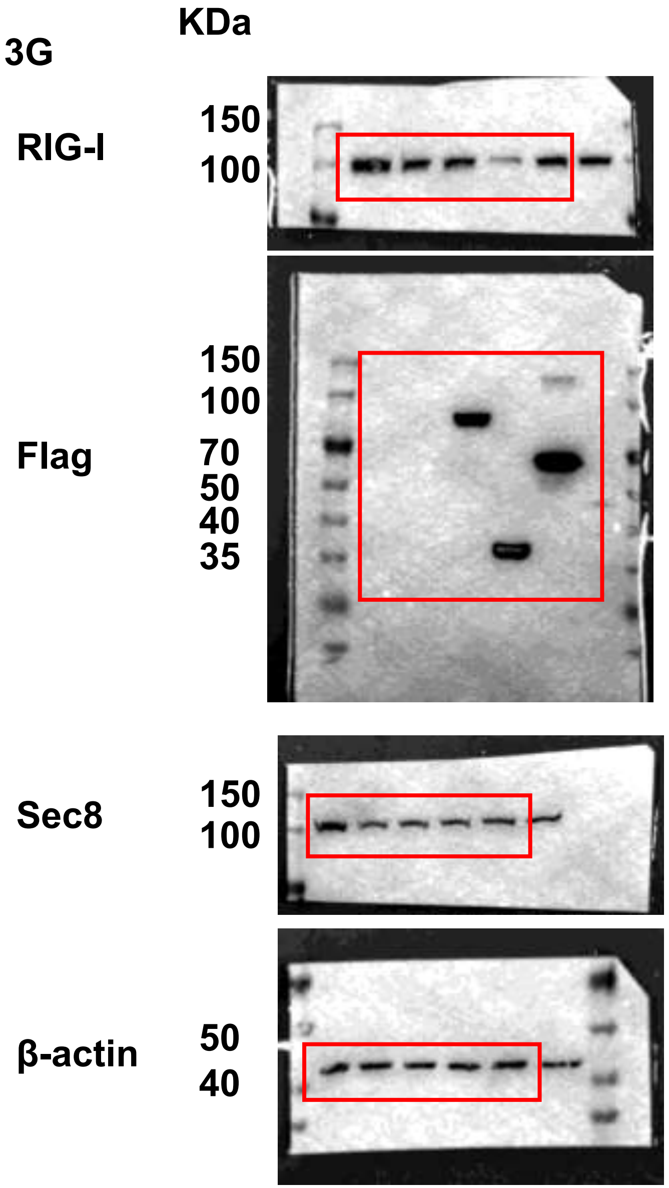

KDa
RIG-I
150
100
Flag
150
100
70
50
40
35
Sec8
150
100
β-actin
50
40

Fig. 3H

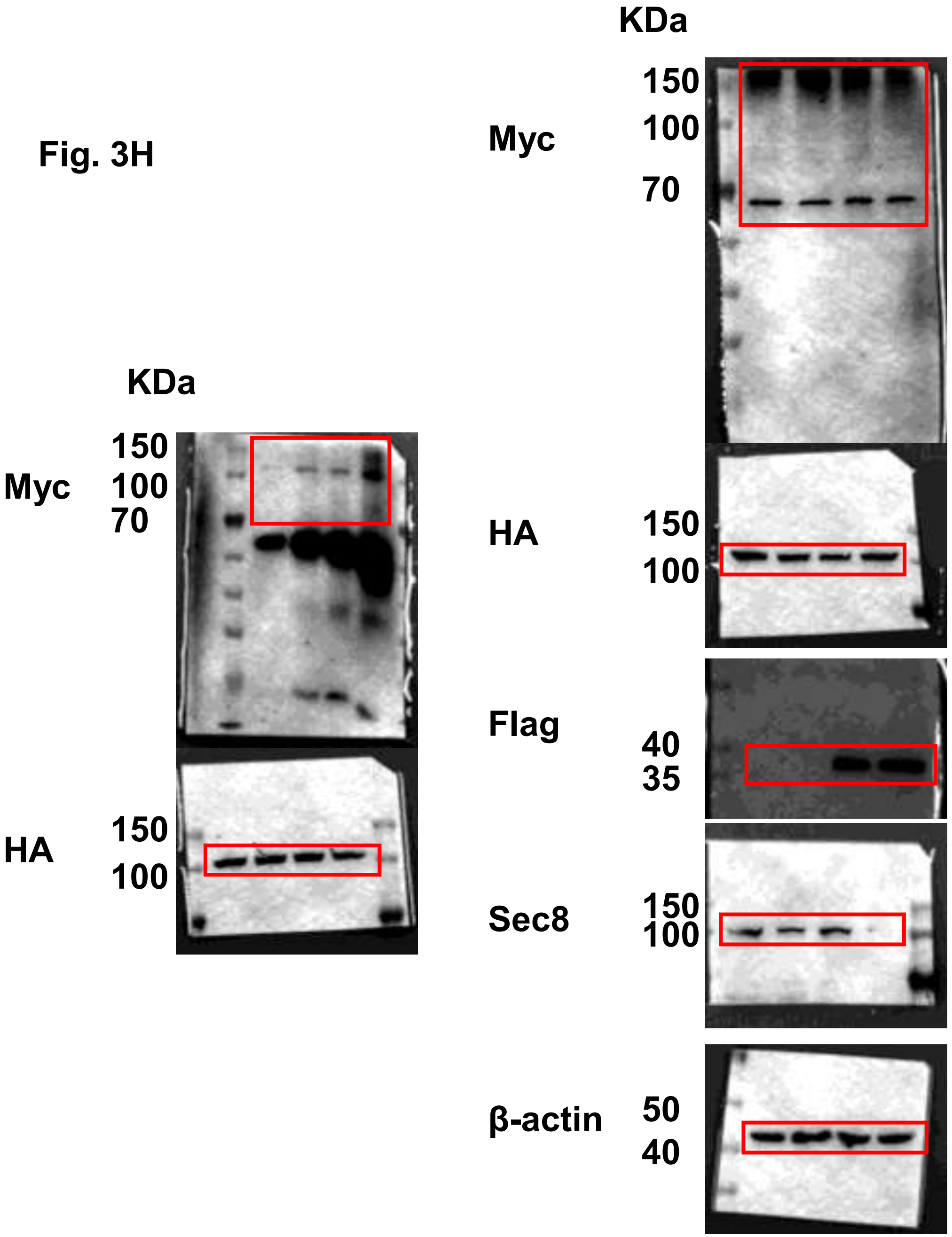

KDa
Myc
150
100
70
HA
150
100
KDa
Myc
150
100
70
HA
150
100
Flag
40
35
Sec8
150
100
β-actin
50
40

Fig. 3I

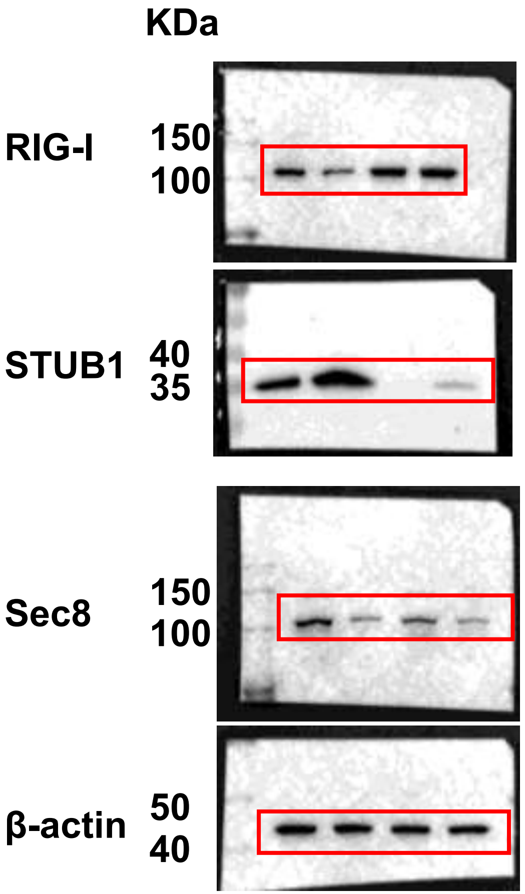

KDa
RIG-I
150
100
STUB1
40
35
Sec8
150
100
β-actin
50
40

Fig. 3J

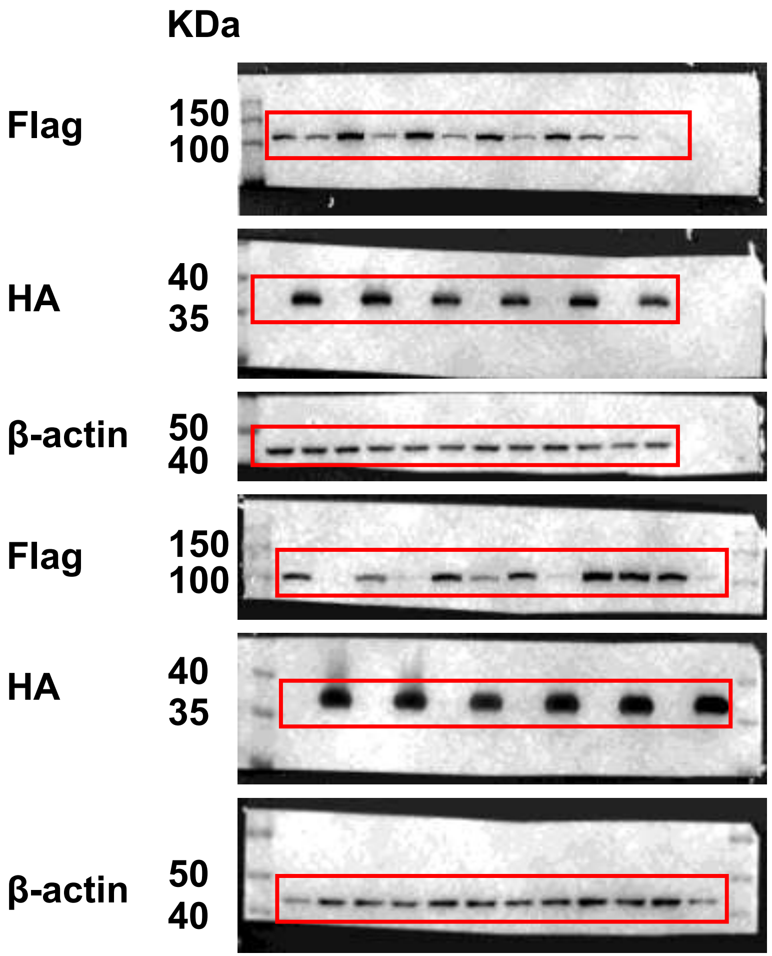

KDa
Flag
150
100
HA
40
35
β-actin
50
40
Flag
150
100
HA
40
35
β-actin
50
40

Fig. 3K

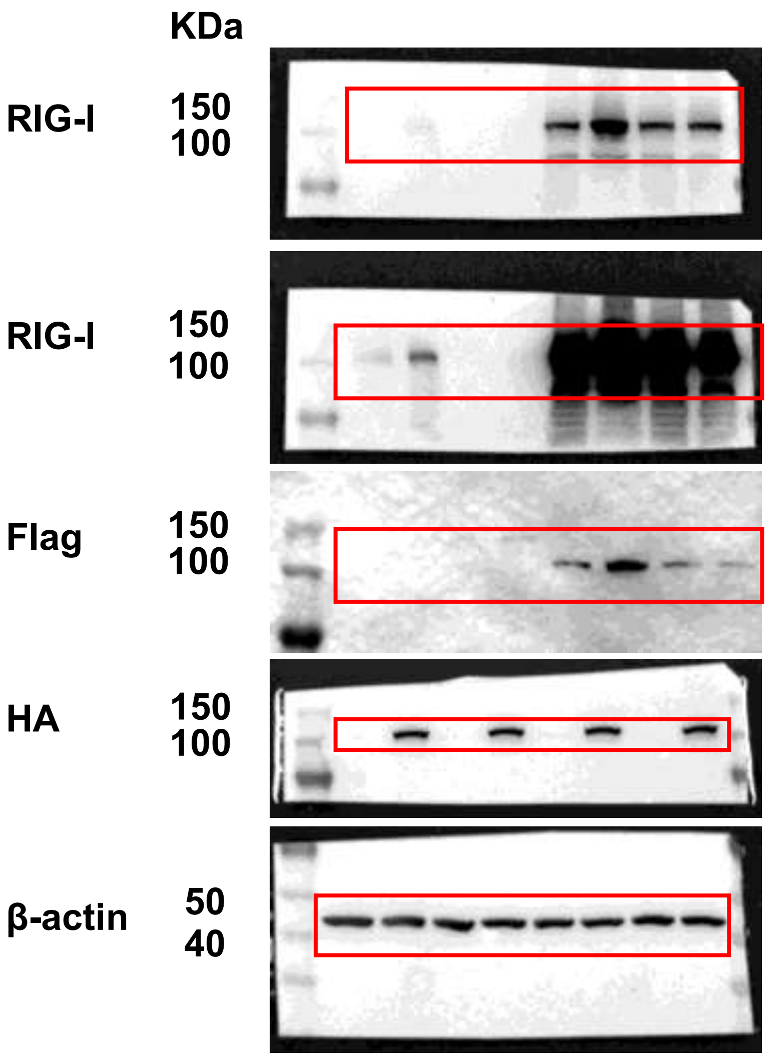

KDa
RIG-I
150
100
RIG-I
150
100
Flag
150
100
HA
150
100
β-actin
50
40

Fig. 4E

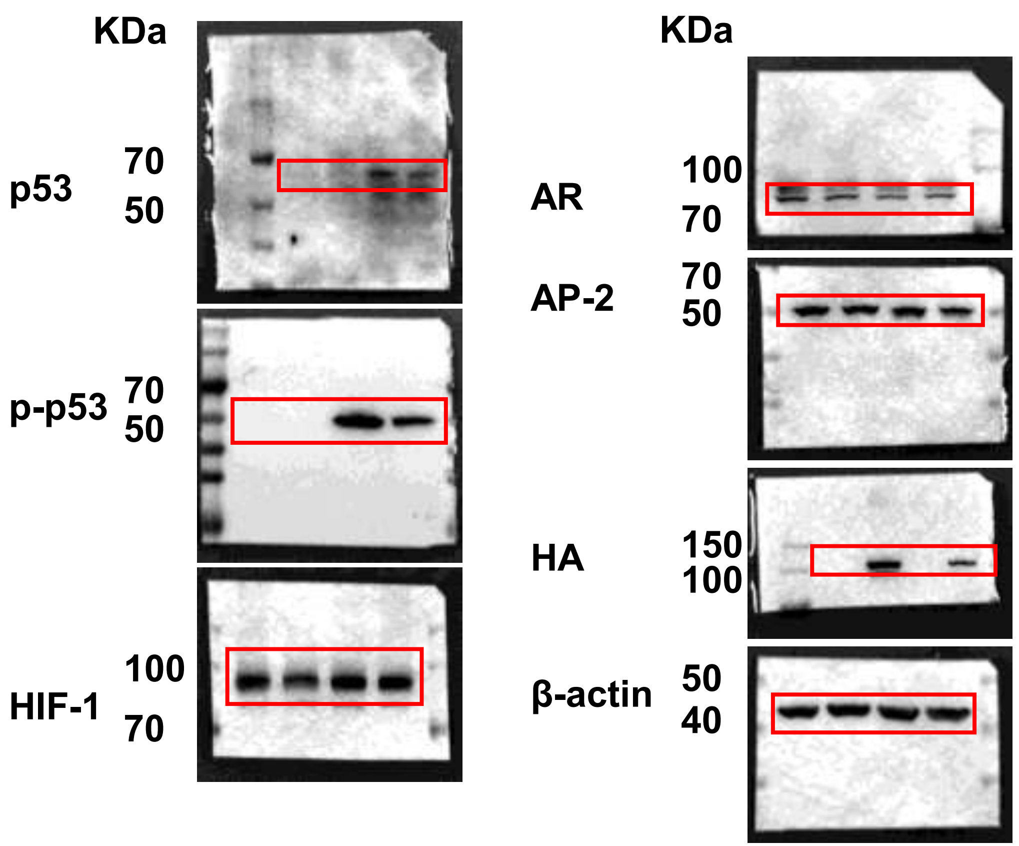
KDa
p53
70
50
p-p53
70
50
HIF-1
100
70
KDa
AR
100
70
AP-2
70
50
HA
150
100
β-actin
50
40

Fig. 4F

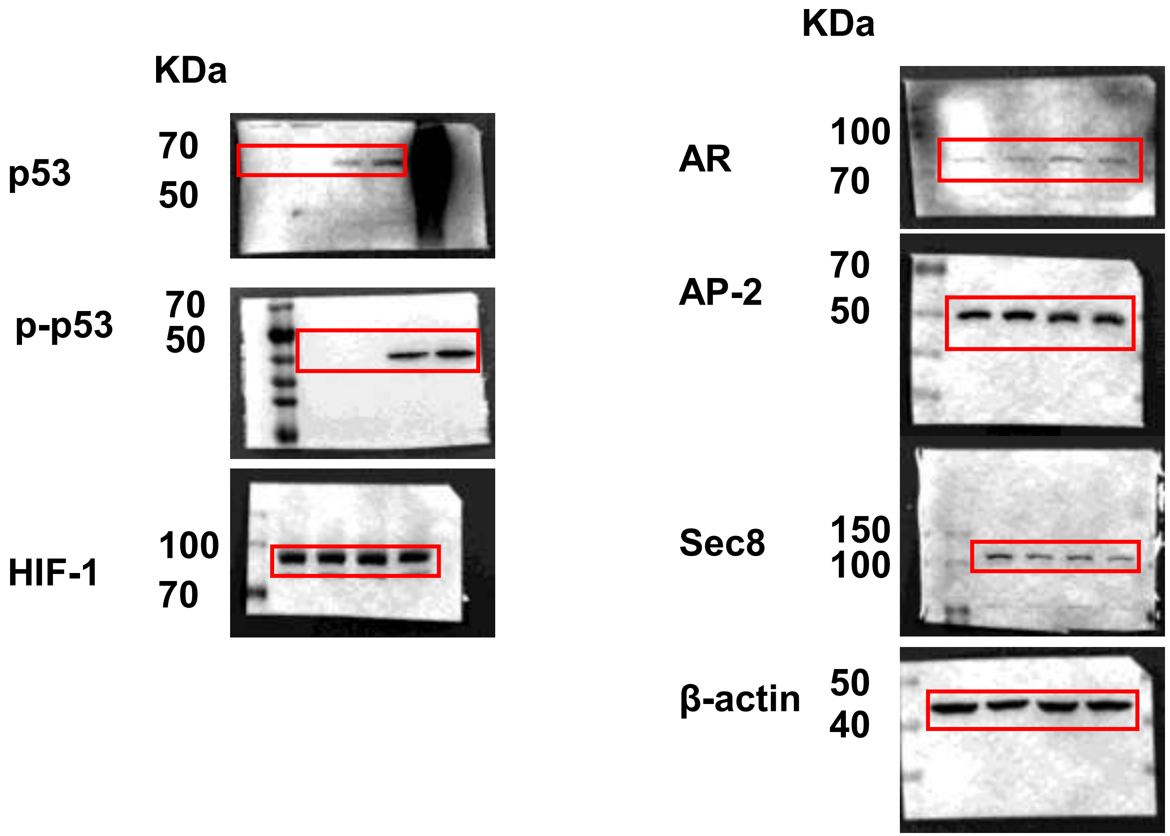

KDa
p53
70
50
p-p53
70
50
HIF-1
100
70
KDa
AR
100
70
AP-2
70
50
Sec8
150
100
β-actin
50
40

Fig. 4H

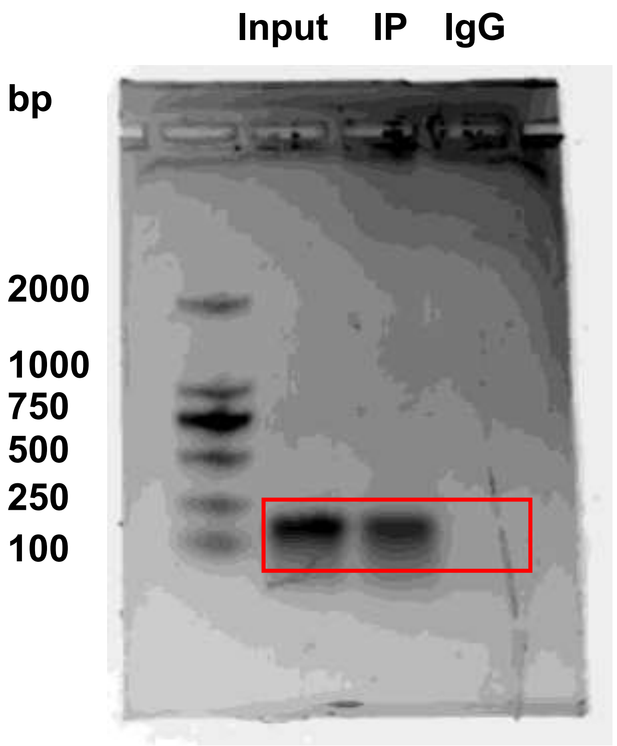
Input IP IgG
bp
2000
1000
750
500
250
100

Fig. 4J

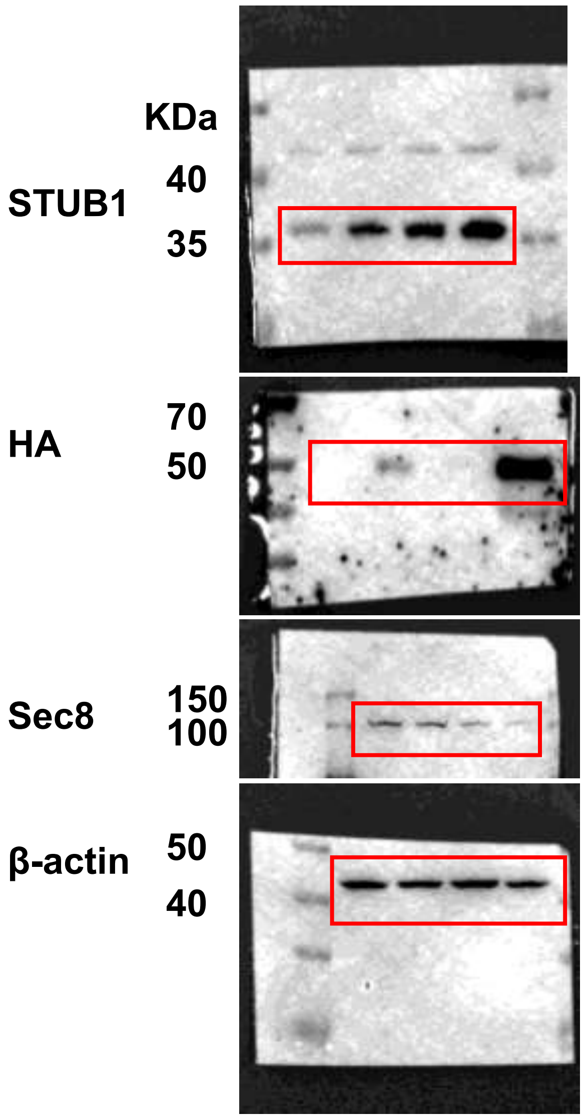
KDa
STUB1
40
35
HA
70
50
Sec8
150
100
β-actin
50
40

Fig. 4K

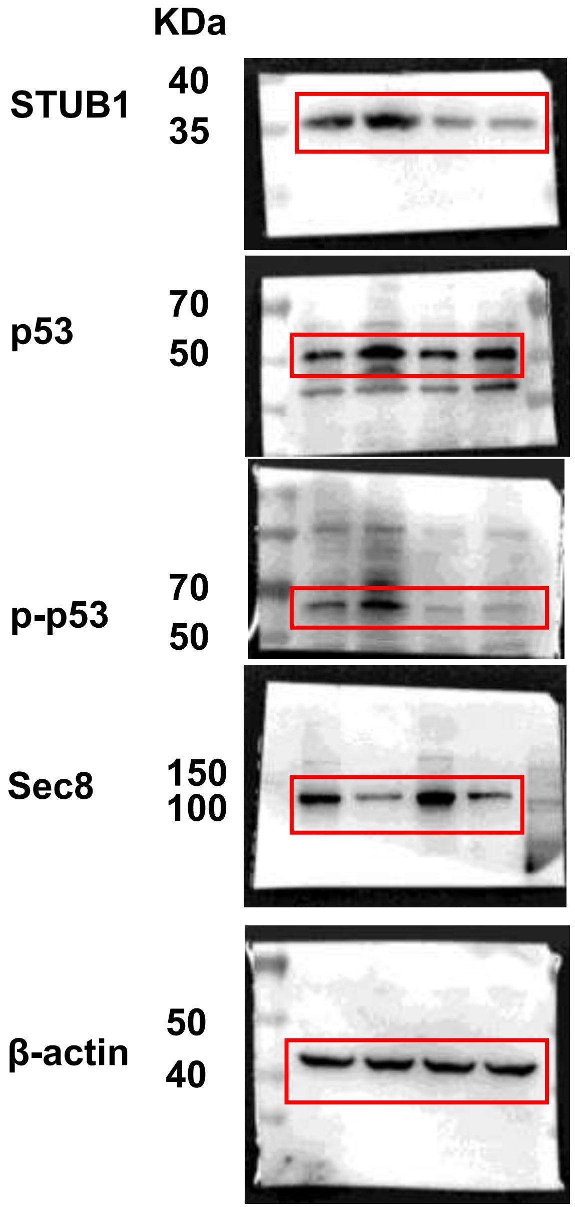

Fig. 5A

Fig. 5B

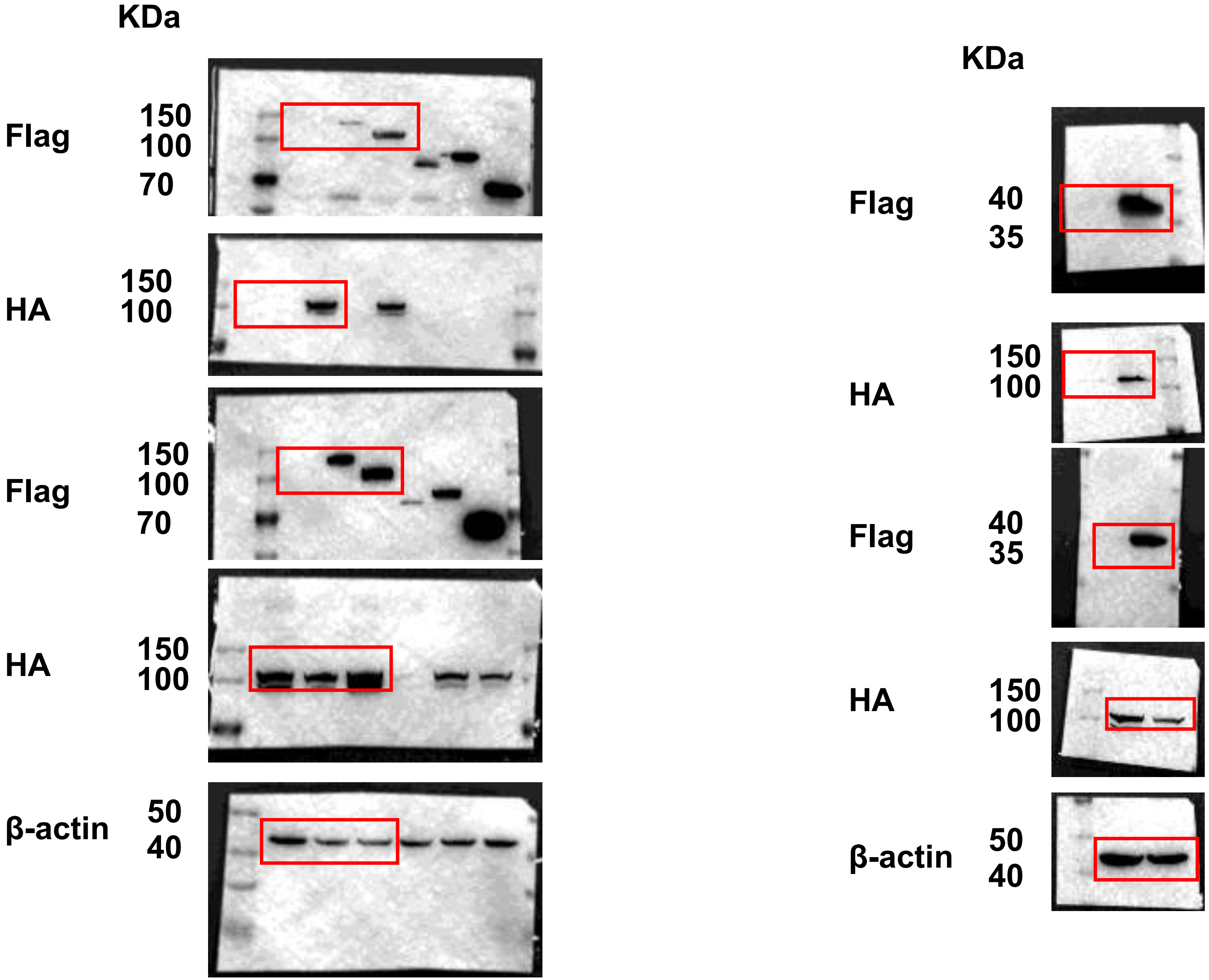

KDa
Flag
150
100
70
HA
150
100
Flag
150
100
70
HA
150
100
β-actin
50
40
KDa
Flag
40
35
HA
150
100
Flag
40
35
HA
150
100
β-actin
50
40

Fig. 5D

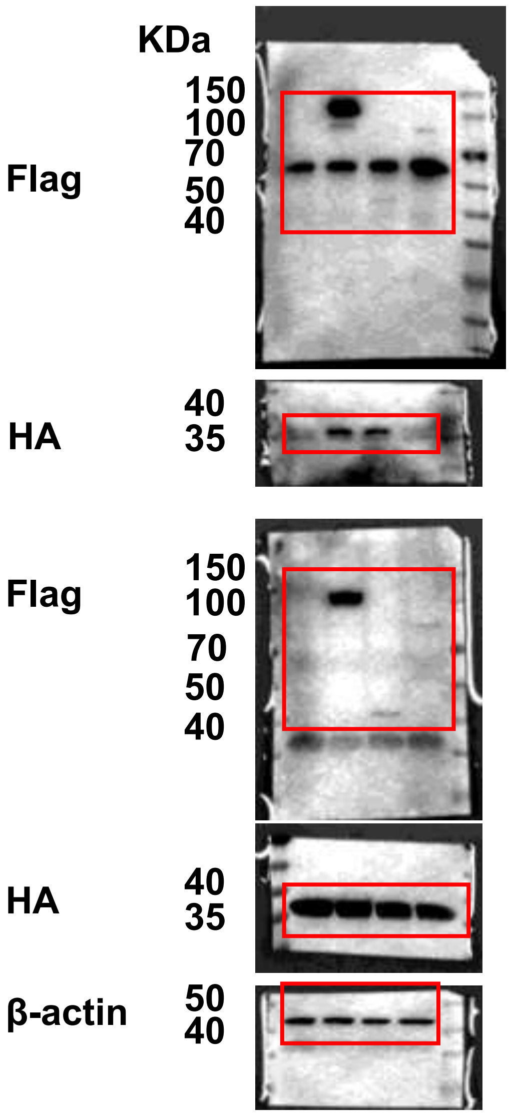
KDa
Flag
150
100
70
50
40
HA
40
35
Flag
150
100
70
50
40
HA
40
35
β-actin
50
40

Fig. 5E

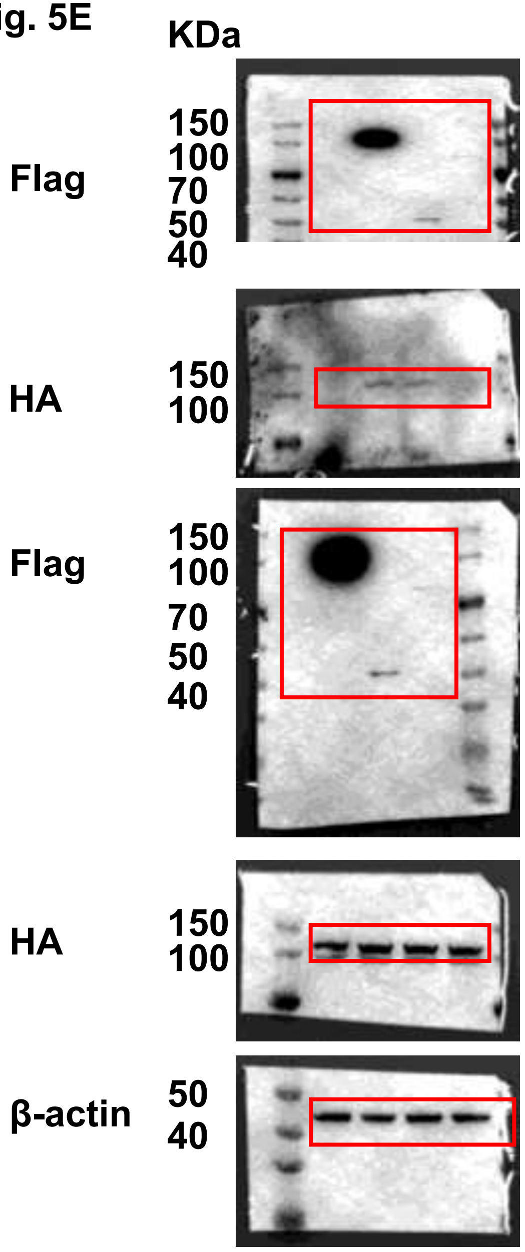
KDa
Flag
150
100
70
50
40
HA
150
100
Flag
150
100
70
50
40
HA
150
100
β-actin
50
40

Fig. 5G

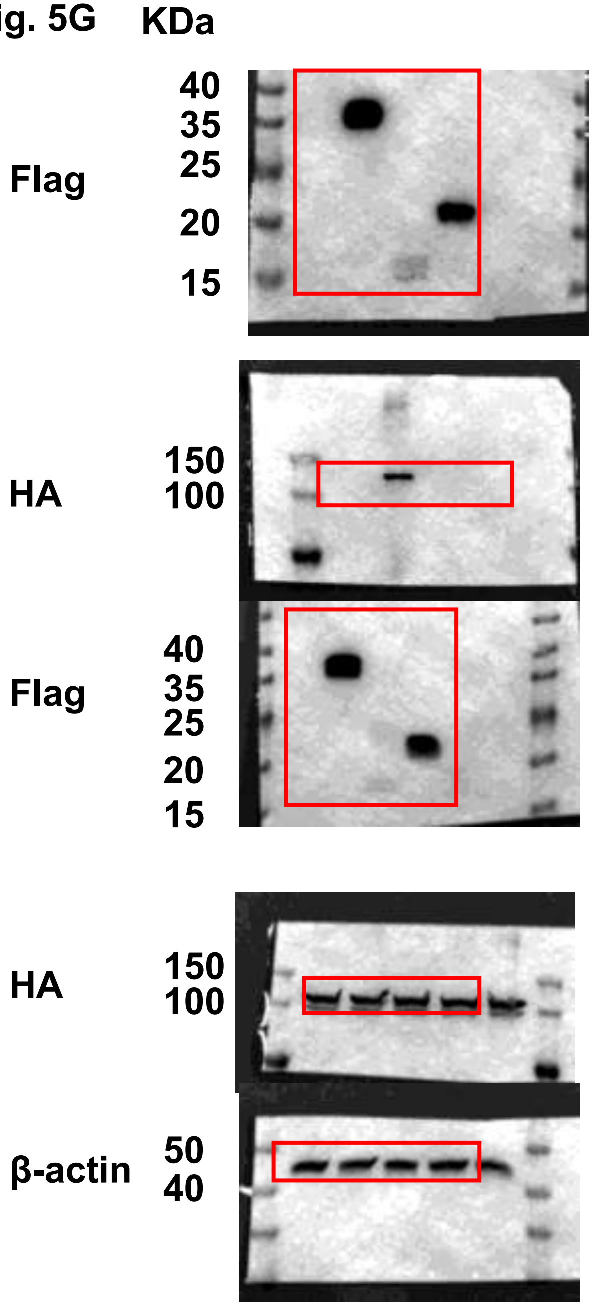
KDa
Flag
40
35
25
20
15
HA
150
100
Flag
40
35
25
20
15
HA
150
100
β-actin
50
40

Fig. 5H

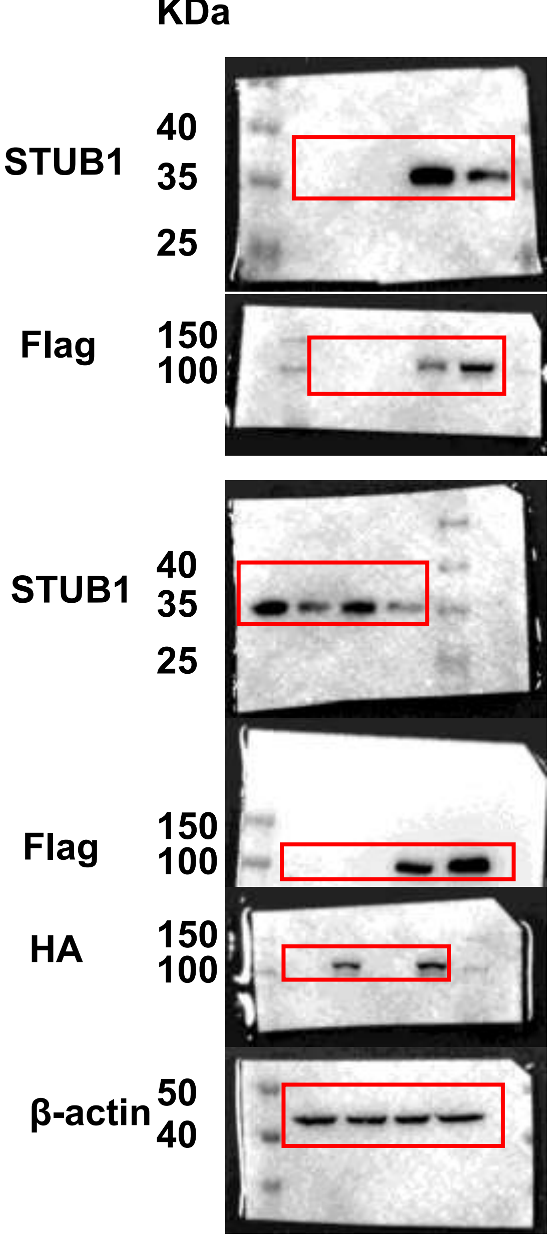
KDa
STUB1
40
35
25
Flag
150
100
STUB1
40
35
25
Flag
150
100
HA
150
100
β-actin
50
40

Fig. 5I

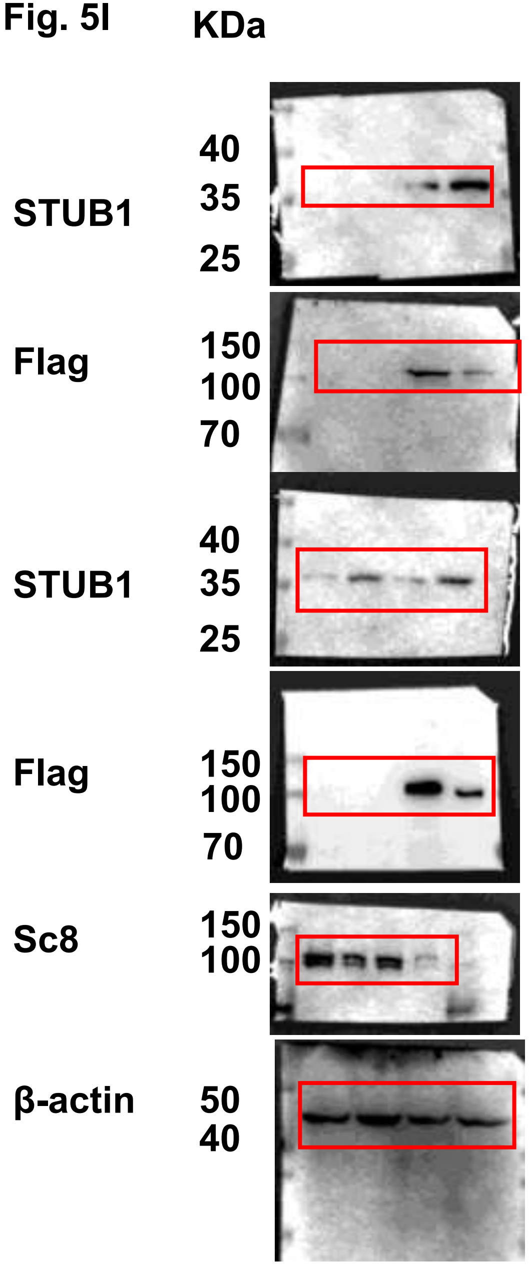
KDa
STUB1
40
35
25
Flag
150
100
70
STUB1
40
35
25
Flag
150
100
70
Sc8
150
100
β-actin
50
40

Fig. 5J

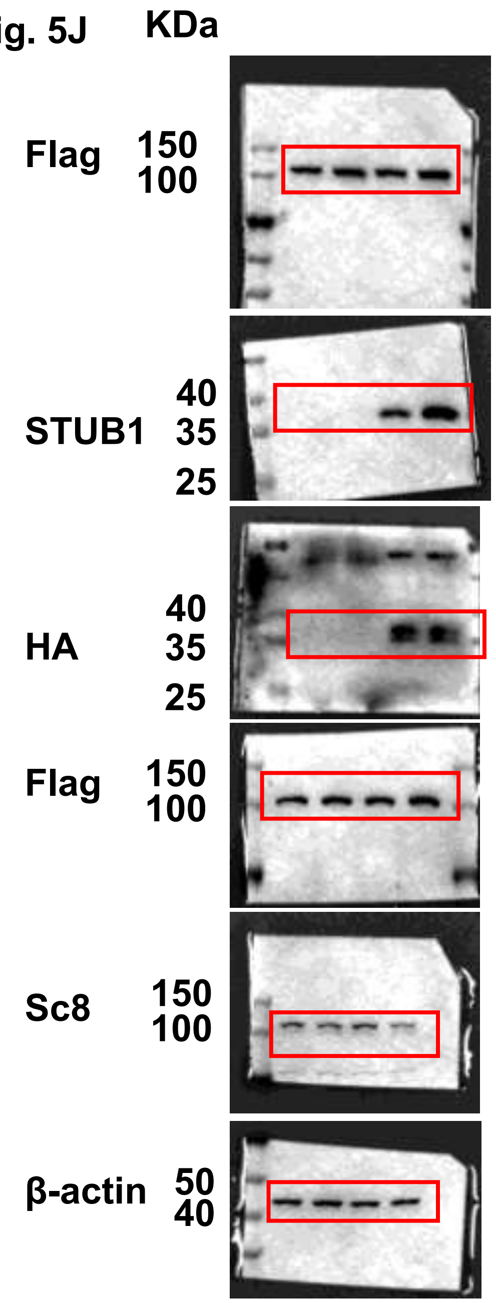
KDa
Flag
150
100
STUB1
40
35
25
HA
40
35
25
Flag
150
100
Sc8
150
100
β-actin
50
40

Fig. S2A KDa

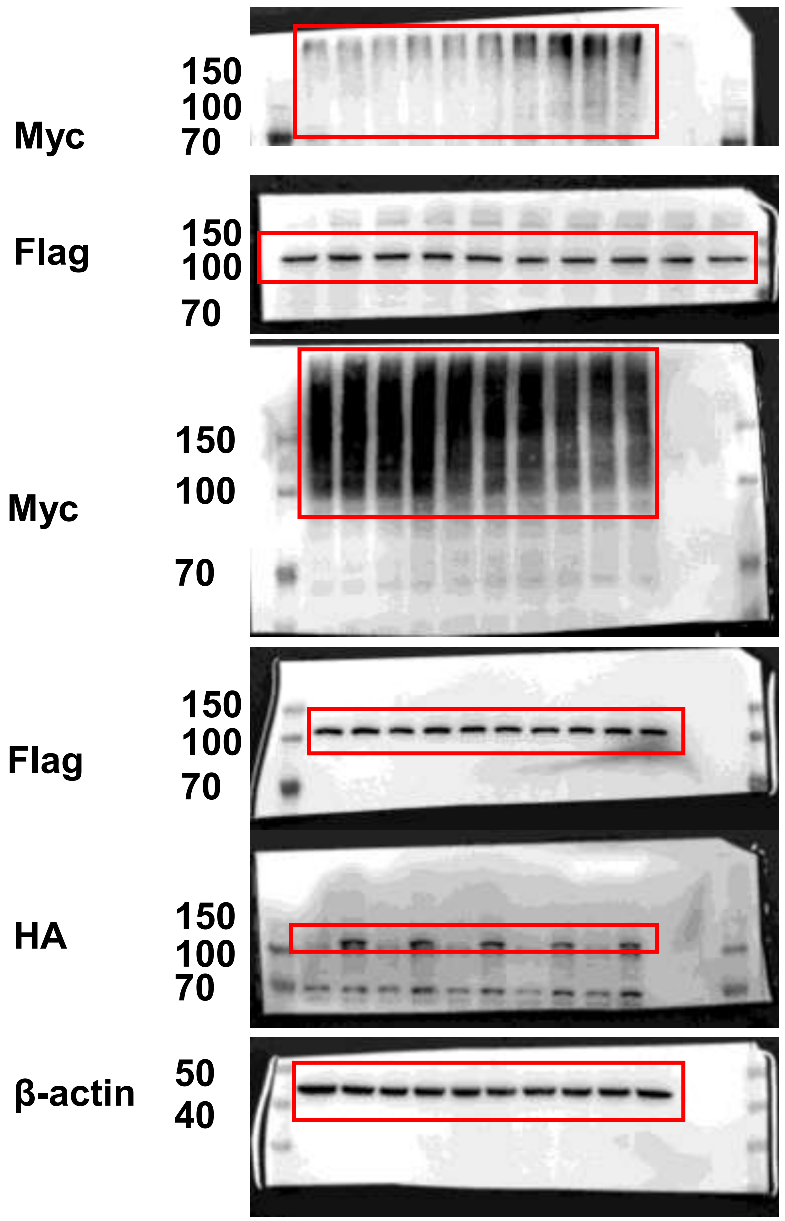

Fig. S2B KDa

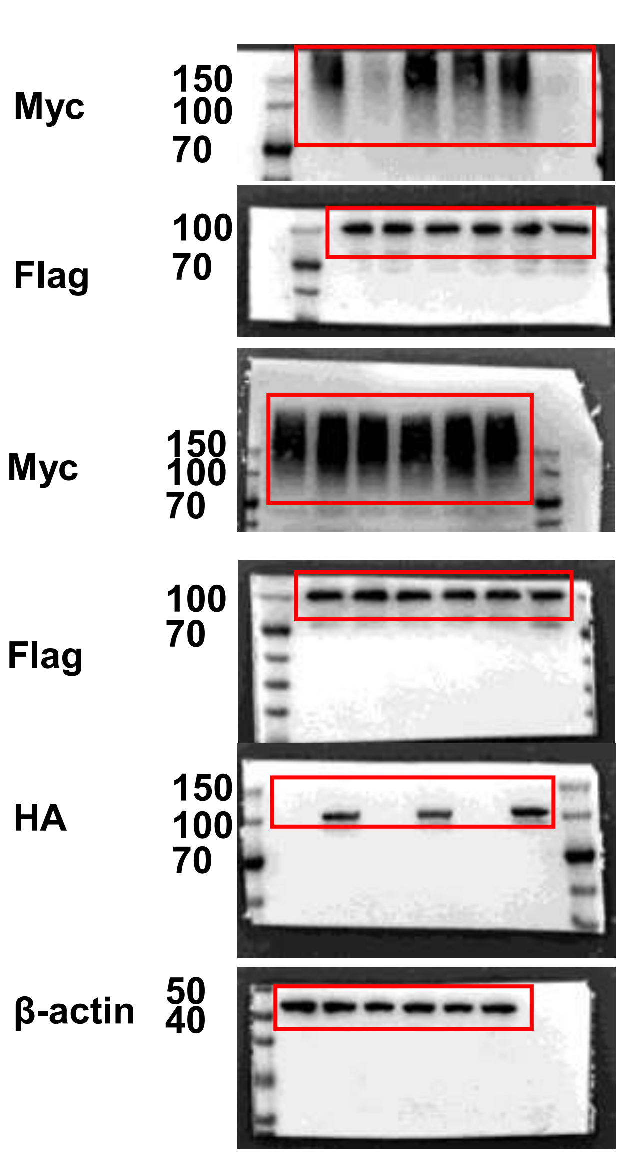

Fig. S2C

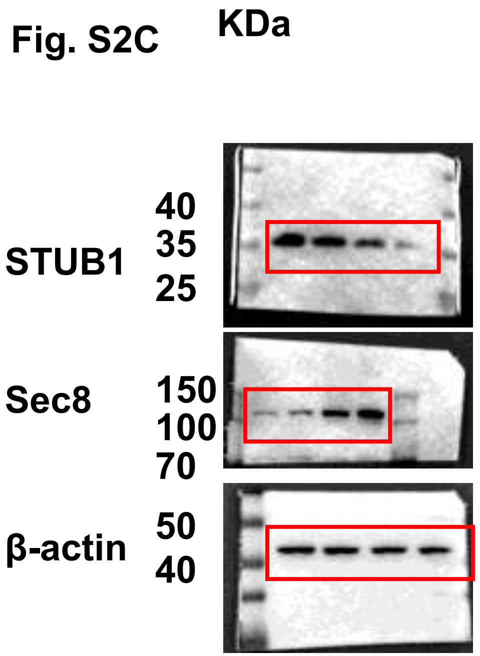

Fig. S2D

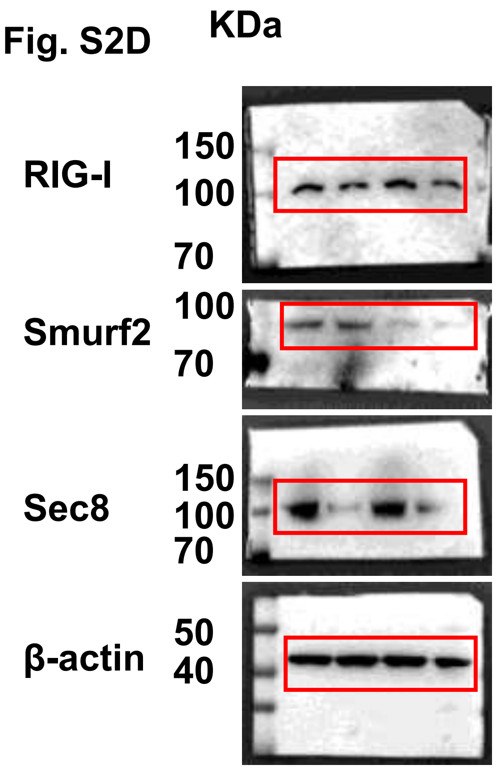

Fig. S2E

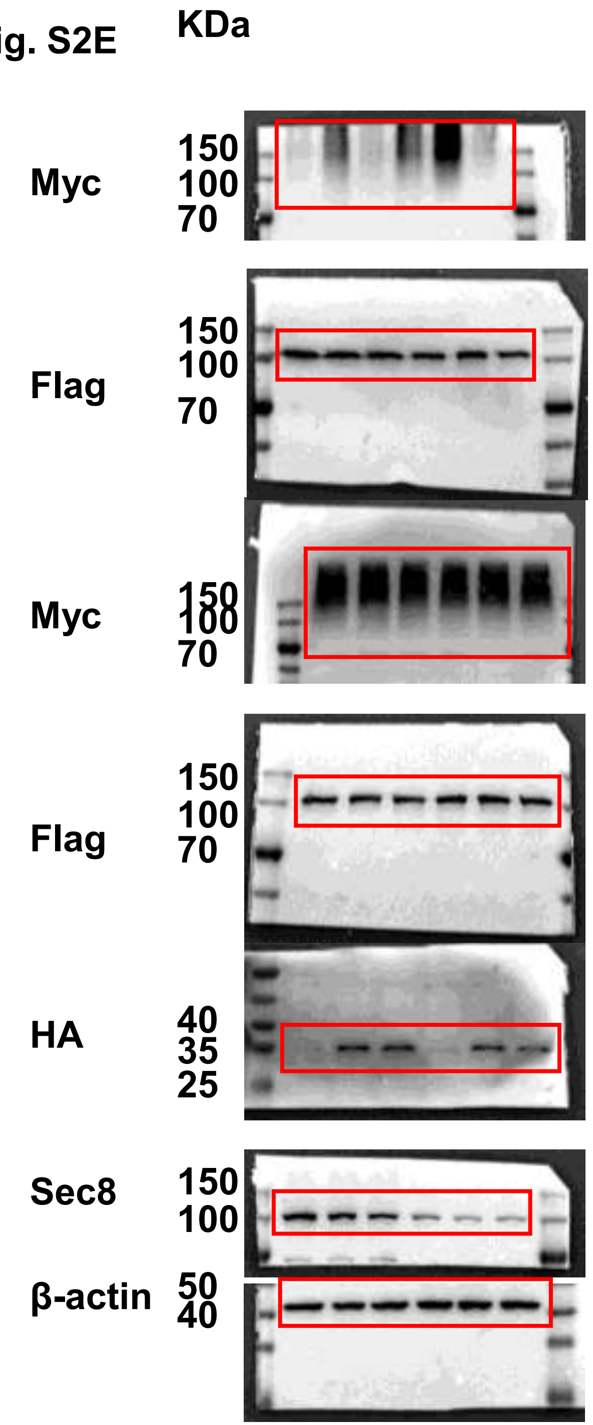

Fig. S3A

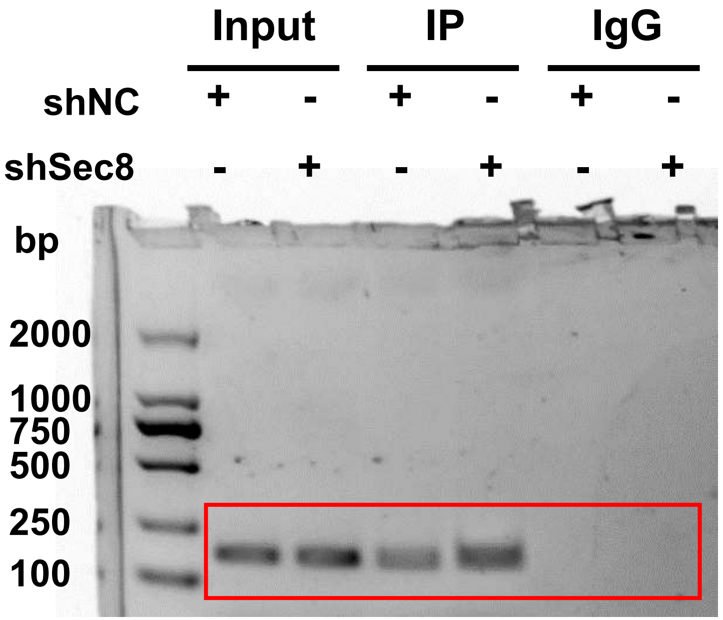

Fig. S3B

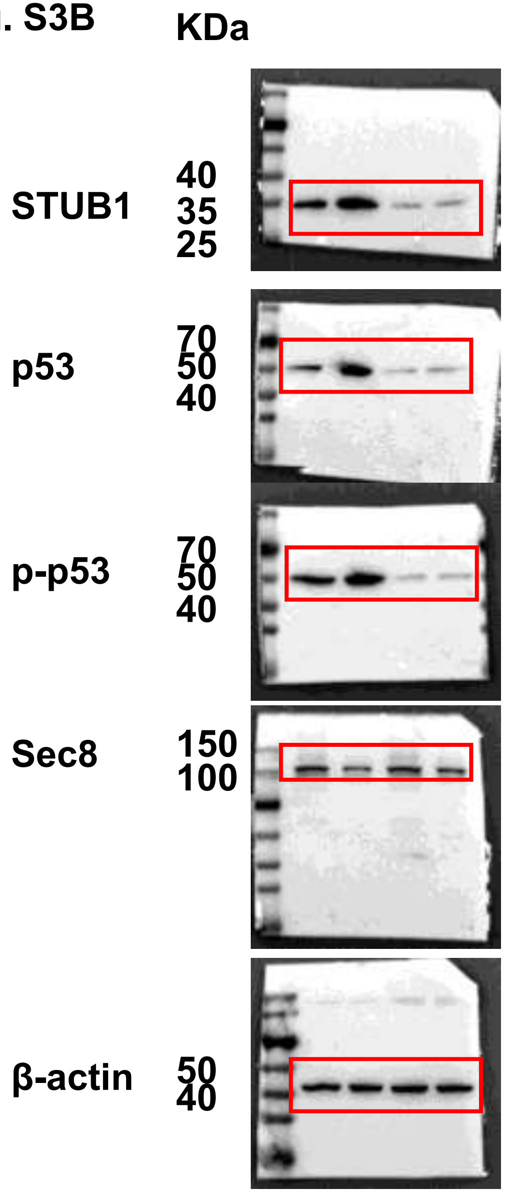

Fig. S4A

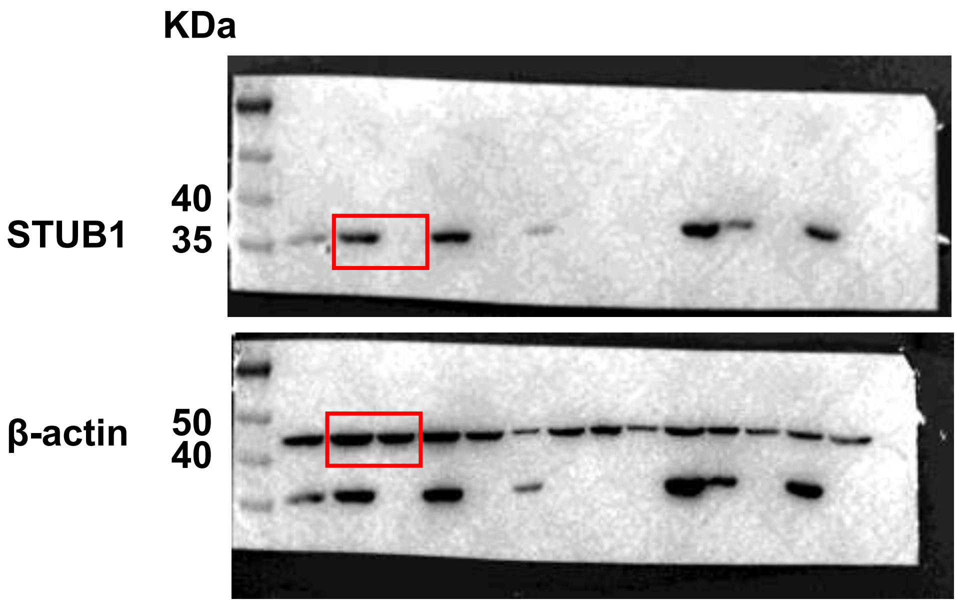

Fig. S4B

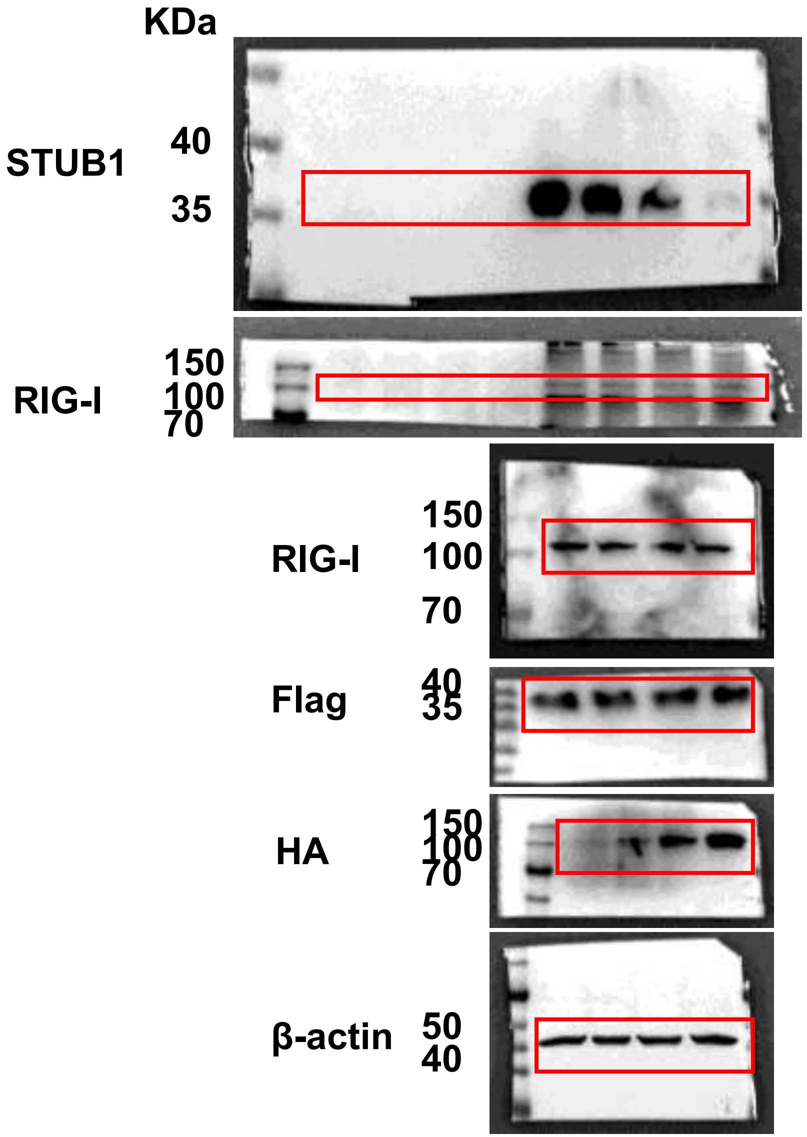

Fig. S4C

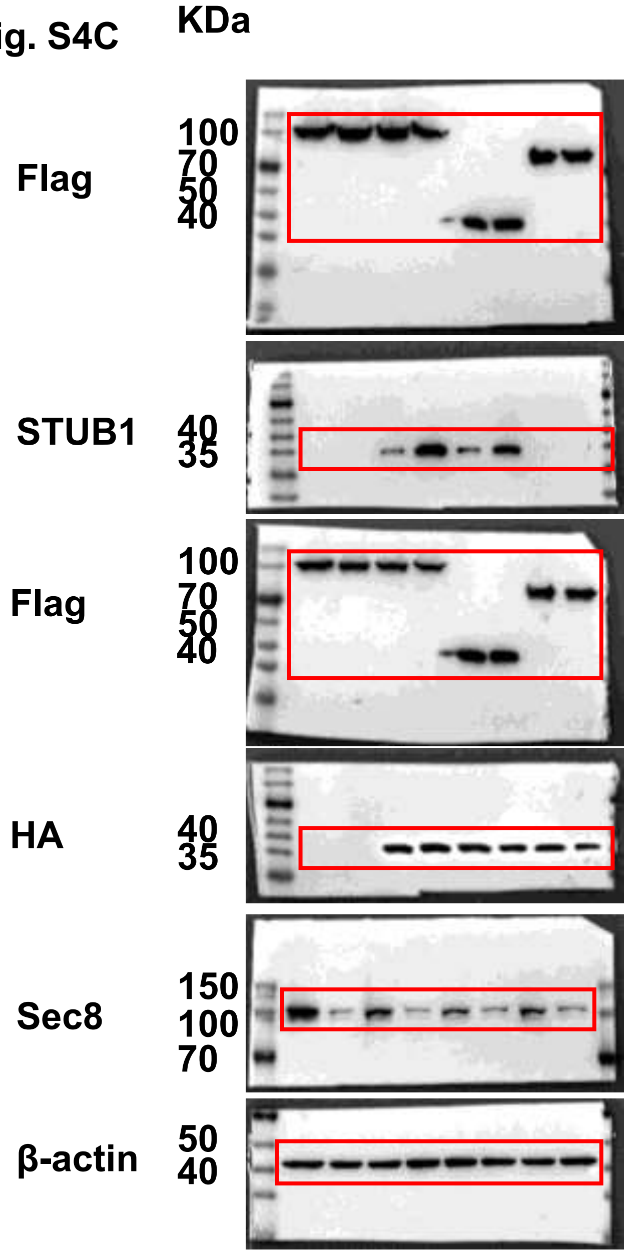
KDa
Flag
100
70
50
40
STUB1
40
35
Flag
100
70
50
40
HA
40
35
Sec8
150
100
70
β-actin
50
40
